# A Model to Derive Langmuir–Hinshelwood Parameters from Heterogeneous Uptake Experiments in a Fast Flow Reactor

**DOI:** 10.1021/acs.jpca.6c03212

**Published:** 2026-08-27

**Authors:** Egor V. Demidov, Alexei F. Khalizov

**Affiliations:** Department of Chemistry and Environmental Science, New Jersey Institute of Technology, Newark, New Jersey 07102, United States

## Abstract

Heterogeneous interactions between reactive trace gases and aerosol particles alter both the atmospheric trace gas composition and the chemical properties of the participating particles. Incorporating these interactions into aerosol models requires kinetic parameters, which are often derived from uptake experiments in flow reactors and are typically limited to uptake coefficients. We present a framework that enables the extraction of a more fundamental set of kinetic parameters, including adsorption, desorption, and reaction rate constants, as well as the concentrations of adsorptive and reactive sites, directly from such uptake data. The framework is currently formulated for the Langmuir–Hinshelwood mechanism on a solid surface but can be readily extended to other mechanisms. The model for time-dependent gas-phase mass transfer and surface chemistry that this framework is based on was developed procedurally, starting from the most rigorous implementation and undergoing two major simplifications, enabling computationally efficient fitting to experimental data. Application of the framework was demonstrated by fitting the model to experimental uptake curves, deriving uptake coefficients and partitioning constants from fitted parameters at both experimental and atmospheric conditions. Finally, an analytical approach for predicting partitioning through reactive Langmuir–Hinshelwood uptake was derived that can improve the accuracy of treating partitioning in aerosol models at no additional computational cost.

## Graphical abstract

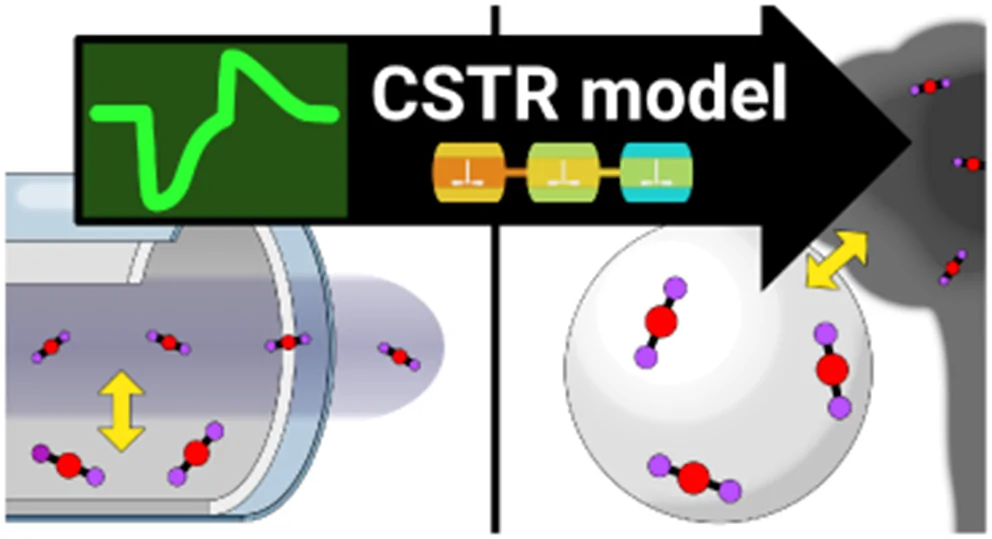

## 1 Introduction

Heterogeneous interactions are major atmospheric processes that involve the uptake of gas-phase reactants by solid or liquid aerosol particles and cloud droplets. Particles and droplets enable reactions that would not have been possible in the gas phase without the presence of a surface or a condensed phase.^1,2^ As a result, the reacting particles and droplets are chemically modified, and atmospheric gas composition is altered. Modification of both the particle and surrounding gas compositions has significant environmental implications. For instance, the heterogeneous uptake of OH radicals on organic aerosol particles substantially alters the climate-forcing properties of those particles by increasing their cloud formation potential. The increase is driven by a rise in hygroscopicity due to an increasing O:C ratio and by changes in their radiative forcing due to a rise in light extinction caused by the modification of the refractive index.^3^ Similarly, the uptake of NO_2_ on mineral dust has been shown to increase particle hygroscopicity by producing hygroscopic nitrate salts on their surface.^4^ In some cases, such chemical processing can suppress cloud formation, as with the heterogeneous uptake of ozone on soot, making particles more hydrophobic.^5^ Examples of environmentally relevant changes in gas-phase composition driven by particle-catalyzed reactions are also abundant. The reaction of gaseous NO_2_ with soot particles occurs at night to produce HONO,^6^ which can serve as a source of OH radicals upon sunrise.^7^ Similarly, at night, N_2_O_5_ is converted into ClNO_2_ through heterogeneous uptake on chloride-containing aerosols.^8,9^ Upon sunrise, ClNO_2_ photolyzes into chlorine radicals, which reduce the atmospheric lifetime of gaseous elemental mercury and hydrocarbons and influence ozone production cycles.^10–12^ The most notorious example of the role of heterogeneous chemistry in the environment is halogen activation on polar stratospheric clouds driven by the heterogeneous uptake of temporary chlorine reservoir species, including HCl and ClONO_2_. Those species engage in a surface reaction, producing gas-phase Cl_2_,^13–15^ which photolyzes into chlorine radicals that deplete ozone upon polar sunrise. Including heterogeneous interactions into atmospheric models requires a formal framework for describing the interaction of gas-phase species with aerosols in a chemical-mechanism-agnostic way, which can be done using either kinetic or thermodynamic approaches.

The kinetic approach simulates heterogeneous uptake as a first-order process with the rate constant *k*_het_ given by

1 $$k_{\mathrm{het}}=\omega A_{v}{[\frac{1}{\Gamma_{\mathrm{diff}}}+\frac{1}{\gamma }]}^{-1}$$

where Γ_diff_ is a parameter that captures the kinetics of diffusion of the gas-phase reactant toward the particle; γ is the uptake coefficient or reaction probability,^16–18^ defined as the ratio of the net uptake flux of the reactant at the surface to the total thermal collision flux of the reactant at the surface; and ω is the mean normal molecular velocity toward the surface:

2 $$\omega =\sqrt{\frac{R_{g}T}{2\pi M}}$$

In the above equation, *R*_g_ is the gas constant, *T* is the temperature, and *M* is the molecular mass. In eq 1, *A*_v_ is the conversion factor between surface and volumetric concentrations. In the case of uptake on aerosol particles, *A*_v_ is the surface area density of particles, having number density *N*_p_ and radius *R*_p_:

3 $$A_{v}=4\pi R_{p}^{2}N_{p}$$

The beauty of this framework is that even without running a model, the uptake coefficient can be used to estimate how quickly gas-phase species will be removed by the surfaces of atmospheric aerosols,^19–22^

4 $$\tau \approx \frac{1}{k_{\mathrm{het}}}$$

where τ is the characteristic lifetime of the gaseous reactant. Not only is this approach chemistry-agnostic, as mentioned above, but it also lumps together in the uptake coefficient information about different stages of the multistep process: adsorption, desorption, surface reaction, and, if applicable, dissolution and bulk reaction. At quasi-steady-state, γ can be approximately deconvoluted into contributions from each step through the resistance model.^23^ For reactive uptake on solid particles (hence, excluding dissolution and bulk reaction kinetics) that follows the Langmuir–Hinshelwood mechanism—a two-step mechanism often used to describe heterogeneous processes— ^24,25^ the resistance model for γ is

5 $$\frac{1}{\gamma }\approx \frac{1}{\gamma_{0}\left(1-\theta \right)}+\frac{1}{\Gamma_{\mathrm{rxn}}}$$

where the term 1/γ_0_(1 – θ) captures the kinetics of site-limited adsorption and the term 1/Γ_rxn_ lumps together the kinetics of desorption and surface reaction; eq 5 is an expression for the uptake coefficient on solid particles derived by Ammann et al.,^24^ where γ_0_ is the initial uptake coefficient of a gas-phase molecule colliding with an unoccupied surface and θ is the Langmuir coverage of adsorbate.

The thermodynamic approach for including heterogeneous chemistry into aerosol models relies on the gas-particle partitioning constant *K*_p_ to describe the gas-particle interactions, and it is widely used to model organic aerosol formation.^26^ The partitioning constant is defined as the ratio of the concentration of the reactant/product in the particle phase to its concentration in the gas phase, normalized by the mass concentration of particles, *P*_m_. It is used to describe equilibrium partitioning in atmospheric models and can give the fraction of the compound present in the particle phase, *f*_p_:^27^

6 $$f_{p}=\frac{K_{p}P_{m}}{1+K_{p}P_{m}}$$

Since *K*_p_ was introduced to model absorption, a mass-controlled process, using it to represent adsorption on solid particles, a surface-controlled process, requires an assumption about the surface-to-mass ratio of particles, which makes it impossible to report a universal, geometry-independent *K*_p_ value for a reactant-substrate pair. Rutter et al.^28^ recognized this limitation of *K*_p_, defined a surface area-normalized gas-particle partitioning constant, and showed that it performed better than *K*_p_ across a broad range of particle sizes:

7 $$K_{\mathrm{sa}}=\frac{K_{p}}{A_{\mathrm{sp}}}$$

where *A*_sp_ is the specific surface area of aerosol particles.

Uptake coefficients and partitioning constants are commonly derived from laboratory experiments. To determine uptake coefficients, among many techniques,^23^ flow reactor experiments are employed for various reactant-surface combinations.^6,19,20,29–33^ In flow reactors, a gaseous reactant is injected into a flow of an inert carrier gas. A segment of the wall of the tubular reactor is coated with a reactive substrate that engages in heterogeneous uptake with the gaseous reactant, acting as a surrogate for an aerosol particle surface. Analysis of flow tube experimental data is often limited to derivation of mechanism-agnostic uptake coefficients from gas-phase reactant rate of loss, which is estimated assuming first-order loss kinetics.^34,35^ Work has been done to relate uptake coefficients to the underlying mechanism, as outlined below.

Before the uptake data can be analyzed, the contribution from the transport of the reactant by advection and diffusion within the reactor needs to be deconvoluted from its loss to the active wall surface. Under fast flow conditions, axial diffusion can be neglected, and when the wall loss is slower than radial diffusion, the contribution from the latter to uptake kinetics in a cylindrical flow reactor can be taken into account through the resistance model^34,36,37^ (eq 1). More recently, Knopf et al.^38^ derived a more accurate method based on explicit analytical equations to account for radial diffusion and the penetration of gas molecules through a cylindrical reactor under laminar flow conditions at steady state. Knopf’s model does not resolve axial concentration gradients but rather considers the gas-wall interactions on average. Gas and surface species concentrations can be resolved along the axial dimension of the reactor using semi-numerical solutions of varying complexity.^39–41^ Correction for diffusion is less straightforward in reactors with complex geometries or when uptake is significantly limited by diffusion, requiring advanced numerical modeling of mass transport in the reactor.^42^

Even after eliminating the contribution from gas diffusion, uptake coefficients do not contain distinct information about adsorption, desorption, and irreversible reaction, but rather they represent lumped combinations of those reaction parameters.^43^ Numerical kinetic models that simulate uptake experiments in a flow reactor have been developed,^44–46^ but none of them can directly invert all of the parameters of the elementary steps of the mechanism from experimental uptake curves. An analytical model by Hanson and Ravishankara^47^ demonstrated that it is possible to extract mechanistic quantities from flow reactor uptake curves. However, the model only extracted a single parameter that lumped together information about transport and reaction and required mechanism-specific analytical solutions.

When predicting heterogeneous uptake on particles in the atmosphere, particle-forward modeling frameworks use parameters either independently constrained from literature, prescribed through assumptions, or extracted from coated reactor or aerosol reactor experiments. To prime the simplest particle-forward models that rely on uptake coefficients, Ammann et al.^24^ derived analytical approximations for uptake coefficients from kinetic parameters of the Langmuir–Hinshelwood mechanism using the resistance model, enabling the prediction of uptake coefficients for the purpose of atmospheric modeling from first principles. Their work established a connection between kinetic parameters of elementary chemical and physical processes and the net uptake coefficient. In later work, the uptake coefficient model was extended to liquid-phase particles, and examples were given to illustrate interactions of gaseous species with aerosols.^48–50^ Their framework does not include a procedure to directly extract kinetic parameters from flow reactor experiments. Instead, they demonstrate the model using a set of independently constrained parameters. Similarly, Wilson and Willis developed models for kinetic uptake on liquid aerosol particles with explicit coupling of vapor-phase and liquid-phase diffusion in a continuum representation.^43,51,52^ Yang et al.^53^ took a different approach, representing reactive uptake stochastically in a Brownian-reactive-dynamics model. Using literature kinetic and thermodynamic inputs, this stochastic model explicitly resolves interfacial and bulk reaction events, demonstrating agreement with continuum models. It should be noted that all of these particle-forward models primarily predict heterogeneous uptake kinetics from prescribed or independently constrained kinetic parameters. They are not formulated to infer elementary parameters directly from the uptake experiments.

Here, we developed a model that resolves gas-phase and surface processes in the domain of a coated wall reactor. The model can be used in two ways: a forward mode to make predictions and an inverse mode to fit kinetic parameters to experimental data. When run in fitting mode, our model can extract the kinetic parameters of elementary steps of the heterogeneous mechanism, which can be used to prime other forward models, e.g., Ammann’s framework for uptake on aerosols. Since gas-phase transport by diffusion and advection in the reactor is represented explicitly, its contribution is automatically excluded from fitted parameters, making them transferable to aerosol models where diffusion kinetics are different. Each extracted parameter provides insight into the physics or chemistry of the corresponding step of the mechanism. We illustrate how kinetic parameters extracted by our model can be used to predict the uptake of a trace gas on aerosol particles by explicitly solving the uptake mechanism equations. We also show how to derive uptake coefficients for higher-level chemistry-agnostic kinetic models using those parameters. Finally, we establish a framework for an equilibrium approach to heterogeneous uptake through partitioning constants calculated from the derived mechanism parameters. A simple analytical model allows us to capture time-dependent reactive uptake within the equilibrium particle representation through an assumed particle lifetime. The connection between elementary parameters of the mechanism and equilibrium constants *K*_p_ and *K*_sa_ enables easy transfer of information obtained with coated reactor experiments to large-scale aerosol models.^54,55^ Presently, the developed model and the derived equations assume that uptake occurs through the Langmuir–Hinshelwood mechanism on a solid surface. However, the model can be extended to other heterogeneous mechanisms, and more complex particle-phase physics and chemistry, like in-particle diffusion, dissolution, and reactions, can also be added in a straightforward manner.

## 2 Model Development

To replicate flow reactor uptake experiments *in silico*, we started with a 2D axisymmetric model to represent gas-phase transport in a cylindrical reactor, allowing us to establish a benchmark with the highest accuracy. Next, we simplified the 2D axisymmetric model to a 1D model by dropping the radial dimension. Finally, we reduced the 1D model to a chained continuous stirred tank reactor (CSTR) model by representing the flow reactor as a system of well-mixed reactors connected in a sequence. The simplifications were necessary to enable inverse runs within a reasonable computational time without compromising model accuracy. In the following sections, we describe each implementation, explain and justify each simplification, and benchmark errors in each simplified model against the 2D model.

To study heterogeneous uptake in a tubular flow reactor, a gaseous reactant is introduced, often via a coaxial injector tube, into a flow of inert carrier gas. A segment of the wall of the reactor coated with a reactive substrate engages in heterogeneous uptake with the gaseous reactant, acting as a surrogate for an aerosol particle surface. The carrier gas and its pressure are usually chosen to maximize the rate of diffusion to ensure that diffusion is not the limiting step in heterogeneous uptake.^20^ Moving the injector axially within the reactor allows us to engage and disengage uptake without interruption of the continuous flow and to carry out repeated experiments. Figure 1 shows four stages of such a heterogeneous uptake measurement. Initially, the injector is extended past the reactive substrate, and none of the gaseous reactant is able to undergo uptake (Figure 1a). The downstream detector measures a constant baseline concentration. To initiate uptake, the injector is retracted so that the reactant mixes with the inert carrier gas before reaching the reactive substrate. As a result, a fraction of the reactant is now able to diffuse toward the substrate and undergo heterogeneous uptake. The detector measures a concentration below the baseline (Figure 1b), and this drop in concentration is a result of reversible adsorption and an irreversible reaction on the surface. As the surface adsorptive sites become saturated, the measured concentration increases to a level just below the baseline level (Figure 1c). At this point, adsorption/desorption is occurring at steady state, and the depressed concentration is caused by reactive consumption of the reactant by the surface. To terminate the run, the injector is extended back to its initial position, where the reactant feed mixes with the carrier gas downstream of the reactive substrate. At this point, the unconsumed reactant, desorbing into the inert carrier gas, combines with the feed, and the detector measures an elevated concentration above the baseline (Figure 1d). When all nonreacted chemicals are desorbed, the concentration measured by the detector returns to the baseline.

**Figure 1:**
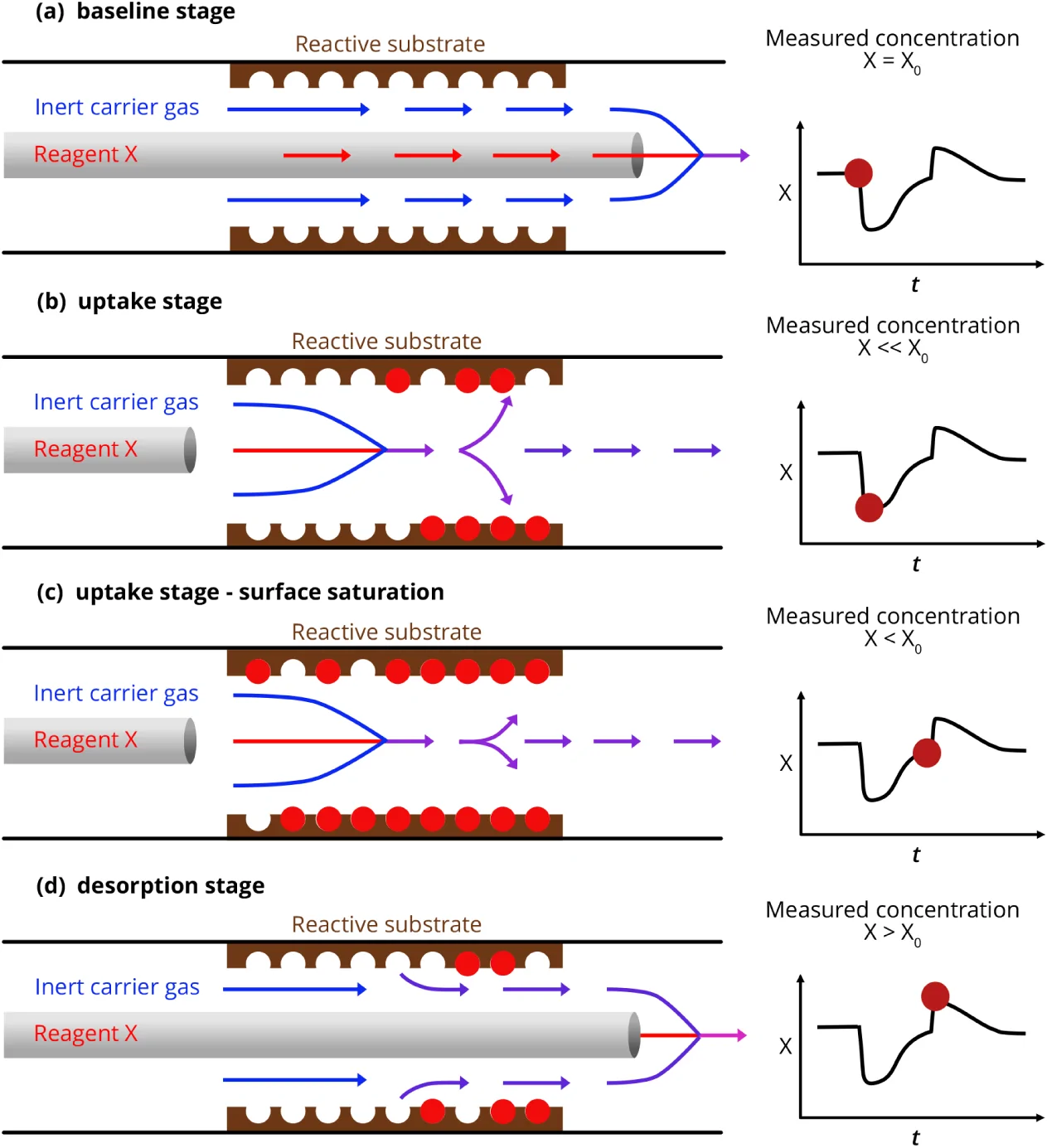
Reaction zone of the tubular flow reactor and four stages of an uptake experiment. By retracting and extending the reactant injector, the reactive substrate can be engaged or bypassed without interrupting the flow: (a) All of the reactant X bypasses the substrate, and baseline concentration is measured by the detector downstream. (b) Reactant X mixes with the carrier gas before reaching the reactive substrate; a fraction of X diffuses toward the substrate and undergoes reactive uptake. The measured downstream concentration is well below baseline. (c) Sorption sites on the surface are saturated, and conversion of the reactant occurs through irreversible reaction on the surface. The measured downstream concentration is slightly below the baseline. (d) Reactant feed bypasses the substrate. Previously adsorbed unreacted X desorbs into the inert gas feed. The measured downstream concentration is above baseline.

For the purpose of this study, we assume that heterogeneous uptake occurs through the Langmuir–Hinshelwood mechanism, which is commonly used to describe the interaction between gaseous reactants and solid surfaces:^24^

8 $$X_{\mathrm{gs}}+S\overset{k_{\mathrm{ads}}}{\underset{k_{\mathrm{des}}}{⇌}}X_{s}$$

9 $$X_{s}+Y\overset{k_{\mathrm{rxn}}}{\to }P+S$$

where species X_gs_ is the gas-phase reactant X in the vicinity of the surface, S are vacant sorptive sites on the surface, species X_s_ is the adsorbed reactant X, Y are vacant reactive sites on the surface, species P is the product of the surface reaction, *k*_ads_ is the adsorption rate constant, *k*_des_ is the desorption rate constant, and *k*_rxn_ is the reaction rate constant. Diffusion of the reactant from the bulk of the flow to/from the wall needs to be either solved for explicitly in a cylindrical domain or accounted for through an additional first-order step:

$$X\overset{k_{\mathrm{diff}}}{↔}X_{\mathrm{gs}}$$ 10

where *k*_diff_ is the diffusion rate constant, which under fast flow conditions in a cylindrical reactor is related to diffusivity *D*_g_ and the reactor radius *R* through^36^

11 $$k_{\mathrm{diff}}=\frac{3.66D_{g}}{R^{2}}$$

The coefficient 3.66 comes from the Gormley–Kennedy solution of radial diffusion in a laminar flow in a cylindrical tube.^56^

In the mechanism described by eqs 8–10, a reactant molecule diffuses toward the surface from the bulk of the gas flow, undergoes reversible adsorption occupying a sorptive site S, and then irreversibly bonds to a reactive site Y to produce product P, releasing the sorptive site S and enabling its reuse by other reactant molecules. A surface fully covered by P is still available for adsorption and desorption through sites S. Occupied and unoccupied surface sites are conserved. From hereon, we adopt the notation where the name of a species is italicized to refer to the concentration of that species. Thus, the balances for sorptive and reactive sites become

$$S_{\mathrm{tot}}=S+X_{s}\;\mathrm{and}\;Y_{\mathrm{tot}}=Y+P$$ 12

where *S*_tot_ and *Y*_tot_ are constants that represent the total concentration of sorptive sites and the total concentration of reactive sites, respectively. A schematic of the Langmuir–Hinshelwood uptake mechanism is shown in Figure 2.

**Figure 2:**
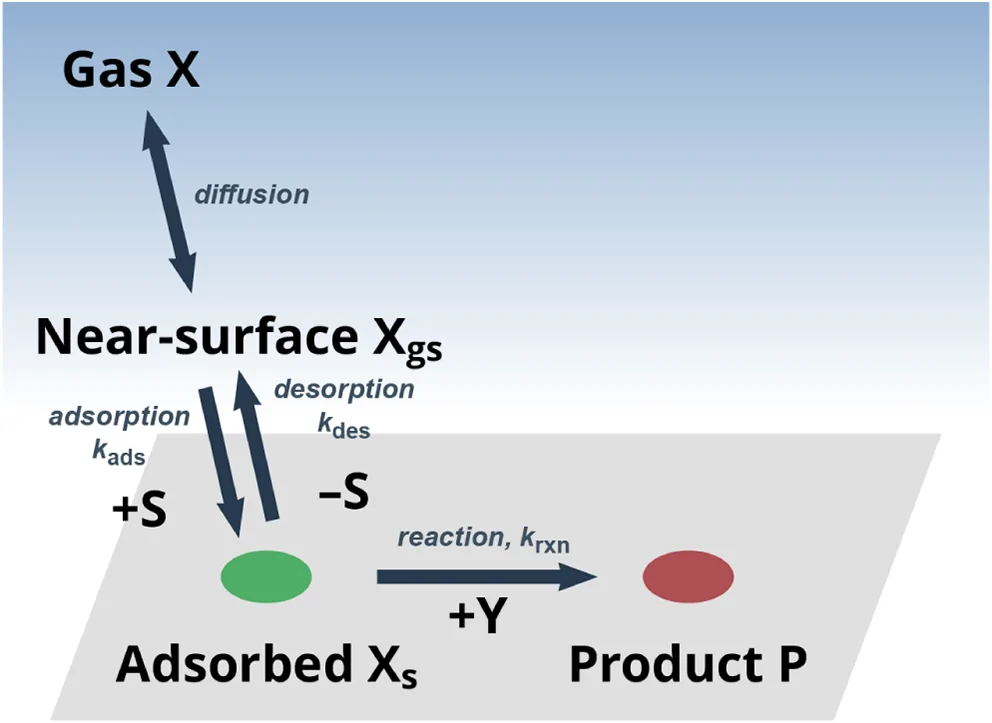
An illustration of the heterogeneous uptake process that follows the Langmuir–Hinshelwood mechanism.

### 2.1 2D Model

To model the transport of a gas-phase reactant X in a tubular flow reactor, advection and diffusion of X need to be considered. The feed of X into the reactor is represented by a Dirichlet boundary condition at the inlet. The mathematical model for X in a laminar flow of gas in cylindrical coordinates is

13 $$\frac{\partial X}{\partial t}+\frac{2F}{\pi R^{2}}\left(1-\frac{r^{2}}{R^{2}}\right)\frac{\partial X}{\partial z}=D_{g}\frac{1}{r}\frac{\partial }{\partial r}\left(r\frac{\partial X}{\partial r}\right)+D_{g}\frac{\partial^{2}X}{\partial z^{2}}$$

where *X* is the gas-phase concentration of the reactant and *F* is the volumetric flow rate of the gas. A laminar flow profile is assumed and is prescribed by the coefficient of *∂X*/*∂z*. Feed concentration of X is prescribed by a Dirichlet boundary condition at the inlet, *X*(r,0,t) = *X*_feed_. A Neumann boundary condition is applied at the centerline to prescribe axial symmetry, ∂*X*(0,*z*,*t*)/∂*r* = 0. The symmetry boundary condition prevents redundancy and allows computation of the solution in one-half of the reactor. Adsorption/desorption of X to/from the wall is represented by a Neumann boundary condition at the reactor wall:

14 $$D_{g}\frac{\partial }{\partial r}X\left(R,z,t\right)=k_{\mathrm{ads}}f_{\mathrm{sw}}\left(t\right)X\left(S_{\mathrm{tot}}-X_{s}\right)-k_{\mathrm{des}}X_{s}$$

Equation 14 describes the gas-surface exchange processes, where the flux of X toward the wall, defined by Fick’s first law of diffusion, is evaluated as the difference between the rates of adsorption and desorption. A switching function *f*_sw_(*t*) simulates the movement of the injector tube, smoothly enabling or disabling the adsorption of X to the surface, as illustrated in Figure 1. The switching function and its parameters are defined as follows:

15a $$k_{\mathrm{sw},1}:=\frac{1}{\tau_{\mathrm{sw},1}},k_{\mathrm{sw},2}:=\frac{1}{\tau_{\mathrm{sw},2}}$$

15b $$a:=\frac{k_{\mathrm{sw},2}}{k_{\mathrm{sw},1}+k_{\mathrm{sw},2}},b:=\frac{k_{\mathrm{sw},1}}{k_{\mathrm{sw},1}+k_{\mathrm{sw},2}}$$

15c $$s:=\frac{1}{k_{\mathrm{sw},2}}\tanh^{-1}\left(\frac{k_{\mathrm{sw},1}-k_{\mathrm{sw},2}}{2k_{\mathrm{sw},1}}\right)$$

15d $$g_{\mathrm{sw}}\left(t\right)=\left\{ \begin{array}{l} a+a\tanh\left(k_{\mathrm{sw},1}\left(t+s\right)\right),t<-s\\ a+b\tanh\left(k_{\mathrm{sw},2}\left(t+s\right)\right),t\geq -s \end{array}\right.$$

15e $$f_{\mathrm{sw}}\left(t\right)=g_{\mathrm{sw}}\left(t-t_{\mathrm{start}}\right)-g_{\mathrm{sw}}\left(t-t_{\mathrm{end}}\right)$$

where *t*_start_ is the nominal time when uptake starts, *t*_end_ is the nominal time when uptake ends, and τ_sw,1_ and τ_sw,2_ are parameters that control the smoothness of transitions. *g*_sw_(*t*) is a piecewise sigmoid function, where the smoothness of the first bend is controlled by τ_sw,1_ and the smoothness of the second bend is controlled by τ_sw,2_. Such a function allows for enabling adsorption with a fast initial jump and smooth leveling off later on. Function *g*_sw_(*t*) is designed to be continuous and to have a continuous first derivative. Physically, τ_sw,1_ and τ_sw,2_ capture the time it takes to retract/extend the injector tube and the time resolution of the detector, controlling the sharpness of peaks on the uptake curve. Parameters *t*_start_ and *t*_end_ are not the times when adsorption starts or ends, but rather the times when *g*_sw_(*t*) = 0.5 and the rate of adsorption is scaled to half of its maximum value.

The concentrations of species *X*_s_ and *P* are modeled with a system of boundary PDEs defined in the one-dimensional reactor wall domain:

16a $$\frac{\partial X_{s}}{\partial t}=k_{\mathrm{ads}}f_{\mathrm{sw}}\left(t\right)X_{\mathrm{gs}}\left(S_{\mathrm{tot}}-X_{s}\right)-k_{\mathrm{des}}X_{s}-k_{\mathrm{rxn}}X_{s}\left(Y_{\mathrm{tot}}-P\right)$$

16b $$\frac{\partial P}{\partial t}=k_{\mathrm{rxn}}X_{s}\left(Y_{\mathrm{tot}}-P\right)$$

where *X*_gs_(*z*,*t*) = *X*(*R*,*z*,*t*). Equations 16a and 21 model surface chemistry that occurs at the wall of the reactor; eq 13 models the transport of X in the gas phase over the entire volume of the reactor; and eq 14, which prescribes a Neumann boundary condition at the wall of the reactor, serves as the link between the gas and wall domains. Initial conditions are *X*(*r*,*z*,0) = *X*_feed_ and *X*_s_(*z*,0) = *P*(*z*,0) = 0.

The main experimental observable is the concentration of gas-phase compound X at the reactor outlet. Since X is radially resolved in the 2D model, the flow-weighted (cup-mixing) average needs to be considered, as it corresponds to the concentration of the effluent stream measured experimentally:

17 $${\mathrm{X̅}}_{\mathrm{out}}\left(t\right)=\frac{4}{R^{2}}\int_{0}^{R}\left(1-\frac{r^{2}}{R^{2}}\right)X\left(r,L,t\right)r\;dr$$

The 2D model, implemented in COMSOL Multiphysics,^57^ takes as input known experimental parameters (reactor length, diameter, flow rate, etc.) and fitted parameters describing the uptake kinetics that are listed in Table 1 along with their units. The five fitted parameters—three rate constants and two surface concentrations—are typically unique for an uptake curve with a complete adsorption–desorption cycle, as discussed in [Sec sec2.4]. The main observable is the radially averaged concentration of X at the outlet as a function of time. If kinetic parameters are chosen appropriately, the plot of concentration vs time will reproduce experimental uptake curves. Figure 3 shows solutions of the 2D model in the gas phase and on the surface at different points during an uptake experiment. Parameters provided in Table 1 were used in these simulations. The fitted parameters in this 2D case were determined manually by trial and error, but an automated fitting procedure was implemented in a simplified version of the model, as discussed in Section 2.3. In experimental inputs, the volumetric flow rate and diffusion coefficient *F*_st_ and *D*_g,st_ are specified at standard pressure. They are converted to absolute volumetric flow rate and diffusion coefficient for use in the model as

18 $$F=\frac{\left(760\;Torr\right)}{P}F_{\mathrm{st}}$$

19 $$D_{g}=\frac{\left(760\;Torr\right)}{P}D_{g,\mathrm{st}}$$

**Table 1:** Parameters Required by the 2D Model, Their Units (T: Time, L: Length, M: Mass), and Whether They Are Known from Experiment or Need to be Obtained from Fitting

Parameter	Symbol	Unit	Type	Example value
Reactor length	*L*	L	known	2 cm
Reactor radius	*R*	L	known	0.78 cm
Volumetric flow rate at st. p.	*F*_st_	L^3^ T^–1^	known	2.5 cm^3^ s^–1^
Pressure	*P*	ML^–1^ T^–2^	known	1.965 Torr
Start of transition smoothness	τ_sw,1_	T	known	1.0 s
End of transition smoothness	τ_sw,2_	T	known	2.5 s
Diffusivity in carrier gas at st. p.	*D*_g,st_	L^2^ T^–1^	known	0.43 cm^2^ s^–1^ ^a^
Feed concentration	*X*_feed_	L^–3^	known	3.0 × 10^9^ cm^–3^
Time uptake starts	*t*_start_	T	known	100 s
Time uptake ends	*t*_end_	T	known	700 s
Adsorption rate constant	*k*_ads_	L^3^ T^–1^	fitted	4.5 × 10^–12^ cm^3^ s^–1^
Desorption rate constant	*k*_des_	T^–1^	fitted	2.0 × 10^–2^ s^–1^
Reaction rate constant	*k*_rxn_	L^2^ T^–1^	fitted	2.5 × 10^–16^ cm^2^ s^–1^
Concentration of adsorptive sites	*S*_tot_	L^–2^	fitted	3.0 × 10^13^ cm^–2^
Concentration of reactive sites	*Y*_tot_	L^–2^	fitted	9.0 × 10^13^ cm^–2^

a Diffusion coefficient of HgCl_2_ in helium at 298 K and 760 Torr.^20^

**Figure 3:**
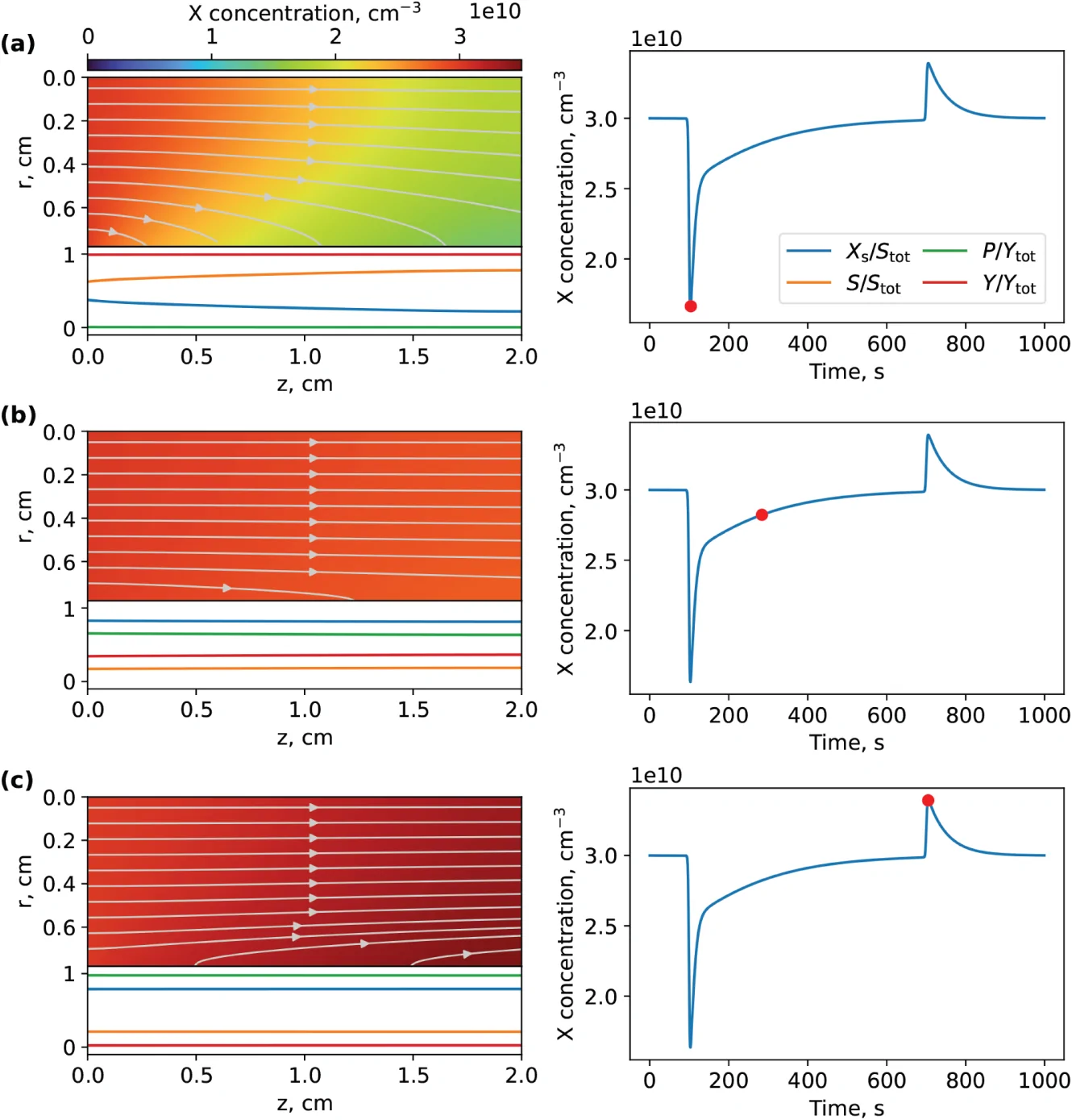
Solutions of the 2D model at different points in time during an uptake experiment. Each section (a–c) of the figure presents: concentration of gas-phase component X over the axial cross-section of the reactor with streamlines indicating the net flux of component X (top left panel); concentrations of surface components *X*_s_, *P, S*, and *Y* along the length of the reactor (bottom left panel); and the generated uptake curve with a dot indicating the time at which the snapshot was taken (right panel). Row (a) corresponds to the start of uptake (Figure 1b); row (b) corresponds to miduptake; and row (c) corresponds to desorption (Figure 1d).

### 2.2 1D Model

The 2D model described in the previous subsection was simplified to a one-dimensional representation of the gas phase, dropping the radial dimension of the reactor. In the 1D version of the model, axial diffusion was still considered explicitly, while radial diffusion was approximated through a first-order rate law (eqs 10 and 11). Surface processes are still modeled with 1D PDEs—eqs 20 and 21 remain unchanged. For the gas phase, eq 13 is replaced by the following system of 1D PDEs:

20a $$\frac{\partial X}{\partial t}+\frac{F}{\pi R^{2}}\frac{\partial X}{\partial z}=-k_{\mathrm{diff}}\left(X-X_{\mathrm{gs}}\right)+D_{g}\frac{\partial^{2}X}{\partial z^{2}}$$

20b $$\frac{\partial X_{\mathrm{gs}}}{\partial t}=k_{\mathrm{diff}}\left(X-X_{\mathrm{gs}}\right)-\frac{2}{R}k_{\mathrm{ads}}f_{\mathrm{sw}}\left(t\right)X_{\mathrm{gs}}\left(S_{\mathrm{tot}}-X_{s}\right)+\frac{2}{R}k_{\mathrm{des}}X_{s}$$

Equations 20 and 21 are unchanged in the 1D model, except that *X*_gs_ is now a separate state representing the concentration near the wall; this eliminates the need to interpolate the 2D solution for *X* at the boundary. The 1D PDE formulation allowed the model to be implemented using an in-house finite difference code.

### 2.3 Chained CSTR Model

The 1D model can be simplified even further into a series of continuous stirred tank reactors (CSTRs). The contents of each reactor are well-mixed and uniform, allowing a reactor to be represented by an ordinary differential equation, significantly reducing the computational burden. For a system of *N*_react_ reactors, the concentration in reactor *i* is given by

21a $$\frac{dX_{i}}{dt}=\frac{FN_{\mathrm{react}}}{\pi R^{2}L}\left(X_{i-1}-X_{i}\right)-k_{\mathrm{diff}}\left(X_{i}-X_{\mathrm{gs},i}\right)$$

21b $$\frac{dX_{\mathrm{gs},i}}{dt}=k_{\mathrm{diff}}\left(X_{i}-X_{\mathrm{gs},i}\right)-\frac{2}{R}k_{\mathrm{ads}}f_{\mathrm{sw}}\left(t\right)X_{\mathrm{gs},i}\left(S_{\mathrm{tot}}-X_{s,i}\right)+\frac{2}{R}k_{\mathrm{des}}X_{s,i}$$

21c $$\frac{dX_{s,i}}{dt}=k_{\mathrm{ads}}f_{\mathrm{sw}}\left(t\right)X_{\mathrm{gs},i}\left(S_{\mathrm{tot}}-X_{s,i}\right)-k_{\mathrm{des}}X_{s,i}-k_{\mathrm{rxn}}X_{s,i}\left(Y_{\mathrm{tot}}-P_{i}\right)$$

21d $$\frac{dP_{i}}{dt}=k_{\mathrm{rxn}}X_{s,i}\left(Y_{\mathrm{tot}}-P_{i}\right)$$

where *i* ∈ [1,*N*_react_], *X*_0_ = *X*_feed_, and *X*_*i*_ at *N*_react_ is the outlet concentration—the main experimental observable.

In addition to the reduction of computational complexity from solving PDEs to solving ODEs, another advantage of the CSTR model is the ability to compute the partial derivatives of species concentrations with respect to fitted parameters exactly (to the extent of the accuracy of the ODE solver). Required for numerical minimization in the process of fitting data, partial derivatives were solved for by augmenting the system of eqs 27 with a system of sensitivity ODEs, which are derived in Section S1 of the Supporting Information. Upon fitting experimental data, uncertainties associated with fitted parameters were calculated using the approach described in Section S2 of the Supporting Information.

The augmented ODE system was solved with the CVODE^58^ solver from the SUNDIALS^59^ library. The Ceres Solver^60^ was used to perform nonlinear fits of simulated uptake curves to experimental data. Two implementations of the CSTR model, one for fitting and the other for forward runs, have been made open source.^61^ A web application providing a user-friendly graphical interface for the CSTR model has been published.^62^

### 2.4 Validation and Applicability Range of the Models

The 2D model is the most physically accurate and can be applied across the broadest range of experimental conditions as long as the gas flow remains laminar, whereas each level of simplification in the other models leads to the omission of certain physics. The 1D model drops the representation of a parabolic flow profile and employs a simplified representation of radial diffusion instead of solving for radial mass transfer explicitly. The CSTR model further drops the representation of axial diffusion and discretizes the axial domain into a series of well-mixed reactors. The simplifications made in the CSTR model allow us to represent the reactor as a system of ordinary differential equations (ODEs) rather than a partial differential equation (PDE) and to solve for sensitivities of the uptake curve with respect to fitted parameters. In this subsection, we establish a quantitative criterion for how many reactors need to be used in the CSTR model to attain reasonable accuracy, and we demonstrate that the 1D and CSTR models perform comparably to the 2D model within the relevant range of experimental conditions.

The error related to discretization of the axial dimension in the CSTR model was assessed by fitting experimental data sets for the uptake of gaseous HgCl_2_ on two different substrates, using different numbers of reactors. One data set contained a nonreactive substrate (levoglucosan), for which only the convergence of parameters *k*_ads_, *k*_des_, and *S*_tot_ is reported; the other data set contained a reactive substrate (NaCl), for which the convergence of *k*_rxn_ and *Y*_tot_ was also tracked. Raw data contained in those experimental data sets and the obtained fitted parameters are provided in Section 3. The convergence of the CSTR solution to the 1D model solution with *N*_react_ ranging from 1 to 40 is visually shown (Figure 4a,c). The relative error of fitted parameters is reported as a function of the number of reactors (Figure 4b,d), treating parameters extracted at *N*_react_ = 40 as the baseline truth. At a high reactor count, the CSTR solution is very close to the 1D model solution.

**Figure 4:**
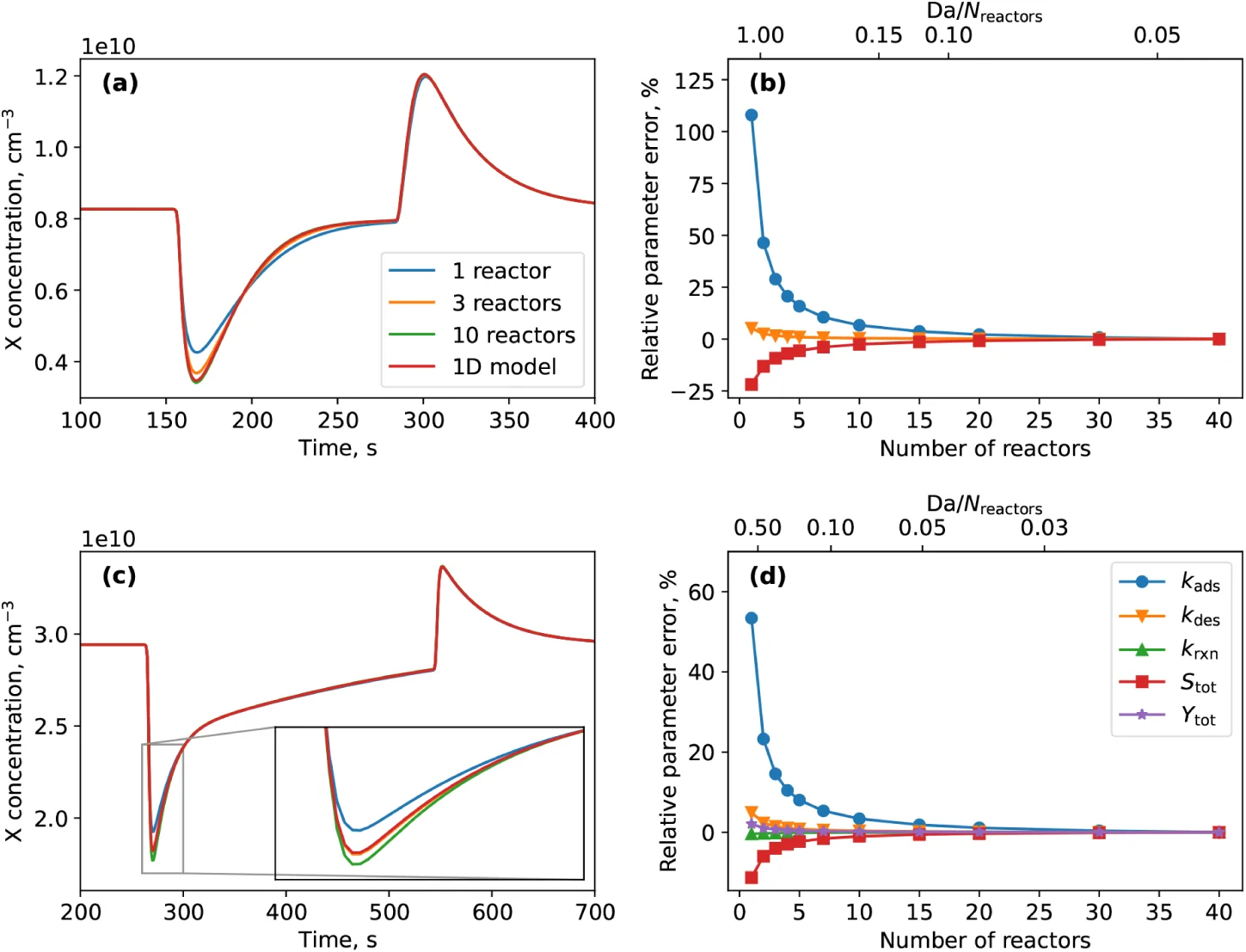
Convergence of the CSTR model with an increasing number of reactors. (a) and (b) Uptake on nonreactive levoglucosan, and (c) and (d) uptake on reactive sodium chloride. Subplots (a) and (c) show that uptake curves generated by the CSTR model approach 1D model curves with an increasing number of reactors. Subplots (b) and (d) show the relative bias of fitted parameters with an increasing reactor count.

We found that the ratio of the Damköhler number of the flow reactor, Da, to the number of chained reactors can be used as a convergence criterion to determine the optimal number of individual CSTR reactors chained together. The Damköhler number is the ratio of flow residence time in the reactor, τ_flow_, to the time scale of the reaction, τ_rxn_. Flow residence time in the reactor is given by

22 $$\tau_{\mathrm{flow}}=\frac{\pi R^{2}L}{F}$$

and a lower bound of the surface reaction time scale can be estimated from the effective first-order rate constant at the beginning of the uptake experiment when *X*_s_ = 0:

23 $$\tau_{\mathrm{rxn}}=\frac{R}{2k_{\mathrm{ads}}S_{\mathrm{tot}}}$$

The CSTR convergence parameter Da/*N*_react_ is then given by

24 $$\frac{\mathrm{Da}}{N_{\mathrm{react}}}=\frac{1}{N_{\mathrm{react}}}\frac{\tau_{\mathrm{flow}}}{\tau_{\mathrm{rxn}}}=\frac{2\pi RLk_{\mathrm{ads}}S_{\mathrm{tot}}}{N_{\mathrm{react}}F}$$

and the condition for accurate representation of a flow reactor with a system of CSTRs is Da/*N*_react_ ≪ 1.

We found from convergence analysis that Da/N_react_ ≈ 0.1 offers the best trade-off between accuracy and performance for the conditions under which the experimental data used in our analysis were obtained. Overall, *k*_ads_ had the highest relative error among other parameters for both nonreactive and reactive substrates. In the case of the nonreactive substrate, limiting the number of reactors to 15 (Da/*N*_react_ ≈ 0.12) biased *k*_ads_ by 3.6%, which is lower than the retrieval uncertainty of 4.2% (Figure 4b). As the Damköhler number varies depending on the geometry of the reactor, flow rate, and the values of fitted parameters *k*_ads_ and *S*_tot_, the optimal reactor count will vary between data sets. For example, in the case of the reactive substrate (Figure 4d), the reactor was shorter (2 cm vs 5 cm for the nonreactive substrate) and Da/*N*_react_ ≈ 0.11 at just 7 reactors, with *k*_ads_ biased by 5.3%, which is less than the 5.5% retrieval uncertainty. When processing an unknown data set, an iterative approach might work best: perform fitting with a smaller number of reactors, evaluate Da/*N*_react_, and increase *N*_react_ until Da/*N*_react_ ≈ 0.15.

Adsorption-related parameters *k*_ads_ and *S*_tot_ have the highest dependence on *N*_react_ because during adsorption, the axial profile of adsorbate *X*_s_ is not uniform and needs to be resolved (Figure 3a). The axial profile becomes uniform only when the entire surface becomes saturated and steady state is reached (Figure 3b). When exposure is terminated and desorption starts, the adsorbate leaves the surface evenly, and the axial profile remains uniform (Figure 3c). As discussed in the sensitivity analysis below, parameters *k*_ads_ and *S*_tot_ are controlled primarily by earlier parts of the uptake curve before steady state is reached, while the parameters *k*_des_, *k*_rxn_, and *Y*_tot_ are determined by the later parts of the curve. A chain of only a few CSTRs does not resolve the axial concentration gradients accurately, failing to reproduce well the part of the uptake curve before steady-state sorption. Thus, fitted parameters *k*_ads_ and *S*_tot_ become biased, leading to a situation where the adsorption rate constant requires axial resolution but the desorption rate constant does not. Reaction-related parameters are even less affected by axial gradients because irreversible reaction is the second, typically slower step of the mechanism. By the time the rate of reaction becomes substantial, sorption has already approached steady state and axial gradients have been leveled out.

To establish the range of experimental conditions under which the simplifications made in 1D and CSTR models remain applicable, we compared the uptake curves generated by these models to those produced by the significantly more rigorous 2D model. The two parameters varied during benchmarking were the feed flow rate and the diffusion coefficient. These were selected because the significance of explicitly accounting for radial diffusion is strongly dependent on the flow rate. In addition, axial diffusion, which is neglected in the CSTR model, can become important at very low flow rates.

To connect the rigorous and approximate benchmarking data sets, the ratio of the radial diffusion time scale to the advection time scale was computed and used as a secondary axis in the generated plots (Figure 5):

25 $$\frac{\tau_{\mathrm{diff},r}}{\tau_{\mathrm{adv}}}=\frac{R^{2}/D_{g}}{L/v_{z}}=\frac{F}{\pi D_{g}L}$$

τ_diff,r_/τ_adv_ is a single parameter to determine whether a simplified model is applicable at a given flow rate and diffusion coefficient.

**Figure 5:**
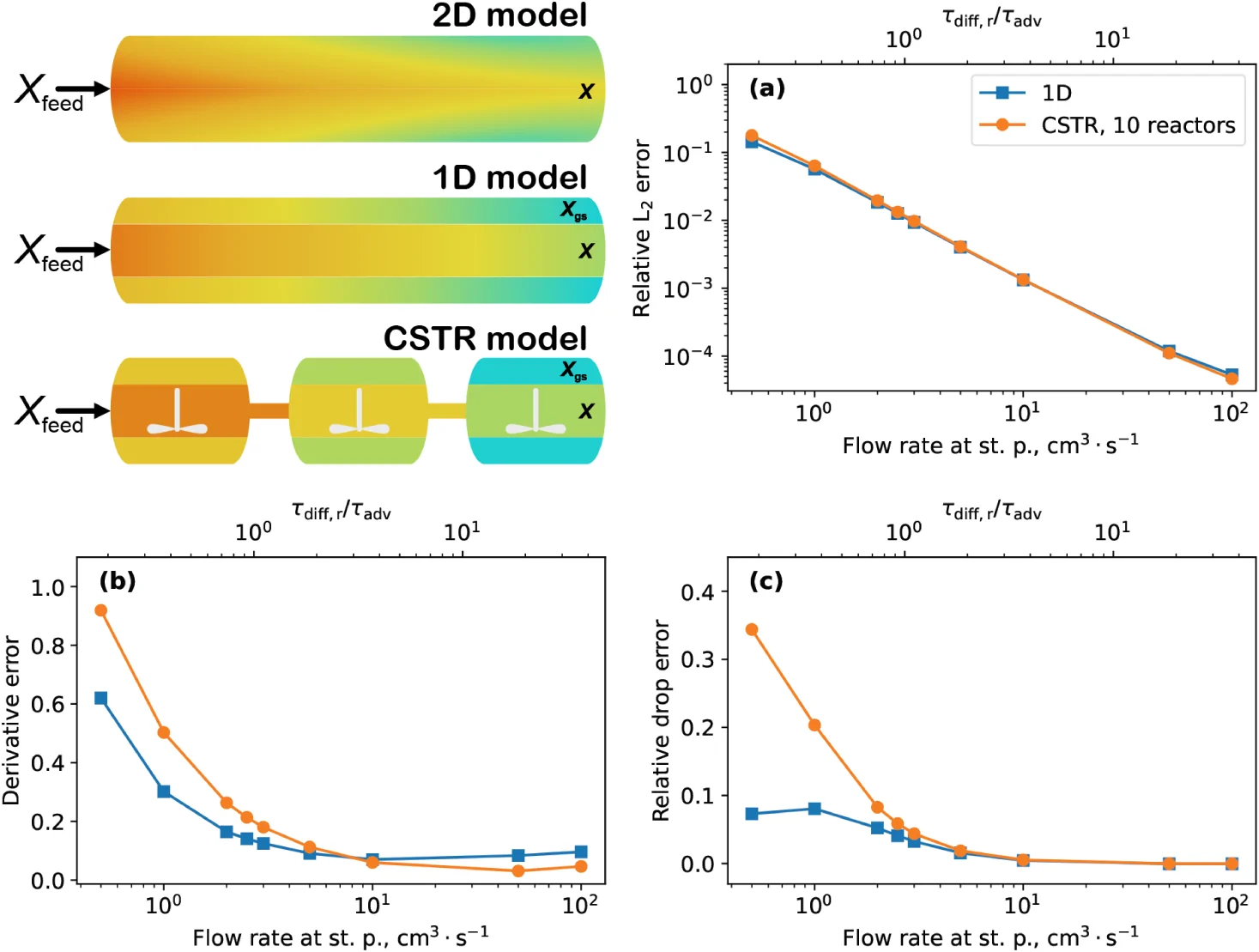
Various metrics of error between the simplified models and the benchmark 2D model at different flow rates for a fixed diffusion coefficient (*D*_g,st_ = 0.43 cm^2^ s^–1^). Top left: illustration showing the level of composition resolution by each model. The 2D model fully resolves axial and radial concentration profiles in the reactor. The 1D model only resolves axial concentration profiles, while the radial concentration profile is binned into two variables: *X* and *X*_gs_. The CSTR model bins radial concentration profiles into the same two variables as the 1D model and bins the axial concentrations into *N*_react_ reactors. Plots (a–c) show solution errors as a function of feed flow rate at standard pressure: (a) Relative *L*_2_ error, (b) derivative error, and (c) relative drop error.

Curves produced by simplified models were compared to curves produced by the 2D model using three metrics: relative L_2_ error, derivative error, and relative drop error. Relative L_2_ error quantifies the squared area between the curves, measuring how well the curves overlap. It is defined as follows:

26 $$E_{L_{2}}=\sqrt{\frac{\int_{0}^{t_{\mathrm{tot}}}{\left(f_{\mathrm{approx}}(t)-f_{\mathrm{true}}(t)\right)}^{2}dt}{\int_{0}^{t_{\mathrm{tot}}}f_{\mathrm{true}}^{2}\left(t\right)dt}}$$

where *f*_true_(*t*) is the uptake curve computed with the 2D model at the given flow rate, *f*_approx_(*t*) is the uptake curve computed either with the 1D model or the CSTR model at the given flow rate, and *t*_tot_ is the duration of the uptake experiment. Derivative error quantifies the squared area between the derivatives of the curves, measuring how well the slopes of the curves match each other. It is defined as follows:

27 $$E_{\mathrm{deriv}}=\sqrt{\frac{\int_{0}^{t_{\mathrm{tot}}}{\left(f_{\mathrm{approx}}^{\prime }(t)-f_{\mathrm{true}}^{\prime }(t)\right)}^{2}dt}{\int_{0}^{t_{\mathrm{tot}}}f_{\mathrm{true}}^{\prime 2}\left(t\right)dt}}$$

where *f*′(*t*) is the first-order derivative of *f*(*t*) with respect to time. Relative drop error quantifies the ratio of the initial drop associated with uptake between the experimental and fitted curves. It is defined as follows:

28 $$E_{\mathrm{drop}}=\frac{\min\left(f_{\mathrm{true}}\left(t\right)\right)-\min\left(f_{\mathrm{approx}}\left(t\right)\right)}{X_{\mathrm{feed}}}$$

Figure 5 shows the results of benchmarking that was performed for the flow rate in the range from 0.5 to 100 cm^3^ s^–1^ with a diffusion coefficient *D*_g,st_ = 0.43 cm^2^ s^-1^, corresponding to the ratio of time scales τ_diff,r_/τ_adv_ from 0.2 to 37. $E_{L_{2}}$ decreases exponentially with the increasing flow rate, changing by 3 orders of magnitude in the benchmarked flow rate range. On the other hand, *E*_deriv_ and *E*_drop_ vary much less, by about an order of magnitude, and converge to their minima after *F*_st_ = 2.5 cm^3^ s^–1^. Accordingly, we conclude that the simplified models produce the lowest error when the feed flow rate is above 2.5 cm^3^ s^–1^ at standard pressure, corresponding to the ratio of time scales above τ_diff,r_/τ_adv_ = 0.9.

Similarly, we benchmarked the model across a range of diffusion coefficients because the need to resolve diffusion explicitly depends on the ratio of the diffusive to advective transport rates.

Figure 6 shows the results of benchmarking that was performed for the diffusion coefficient in the range from 0.01 to 1.0 cm^2^ s^–1^with a flow rate of *F*_st_ = 2.5 cm^3^ s^–1^, corresponding to the ratio of time scales τ_diff,r_/τ_adv_ from 40 to 0.4. $E_{L_{2}}$ and *E*_deriv_ both decrease with increasing diffusion coefficient, reach minima after *D*_g,st_ = 0.05 cm^2^ s^–1^, and increase again after *D*_g,st_ = 0.1 cm^2^ s^–1^. *E*_drop_ starts off slightly negative and increases linearly, going through zero near *D*_g,st_ = 0.05–0.1 cm^2^ s^–1^. Results of benchmarking allow us to conclude that the simplified models produce the lowest error when the diffusion coefficient is in the range 0.05–0.1 cm^2^ s^–1^, corresponding to the ratio of time scales τ_diff,r_/τ_adv_ from 8 to 4.

**Figure 6:**
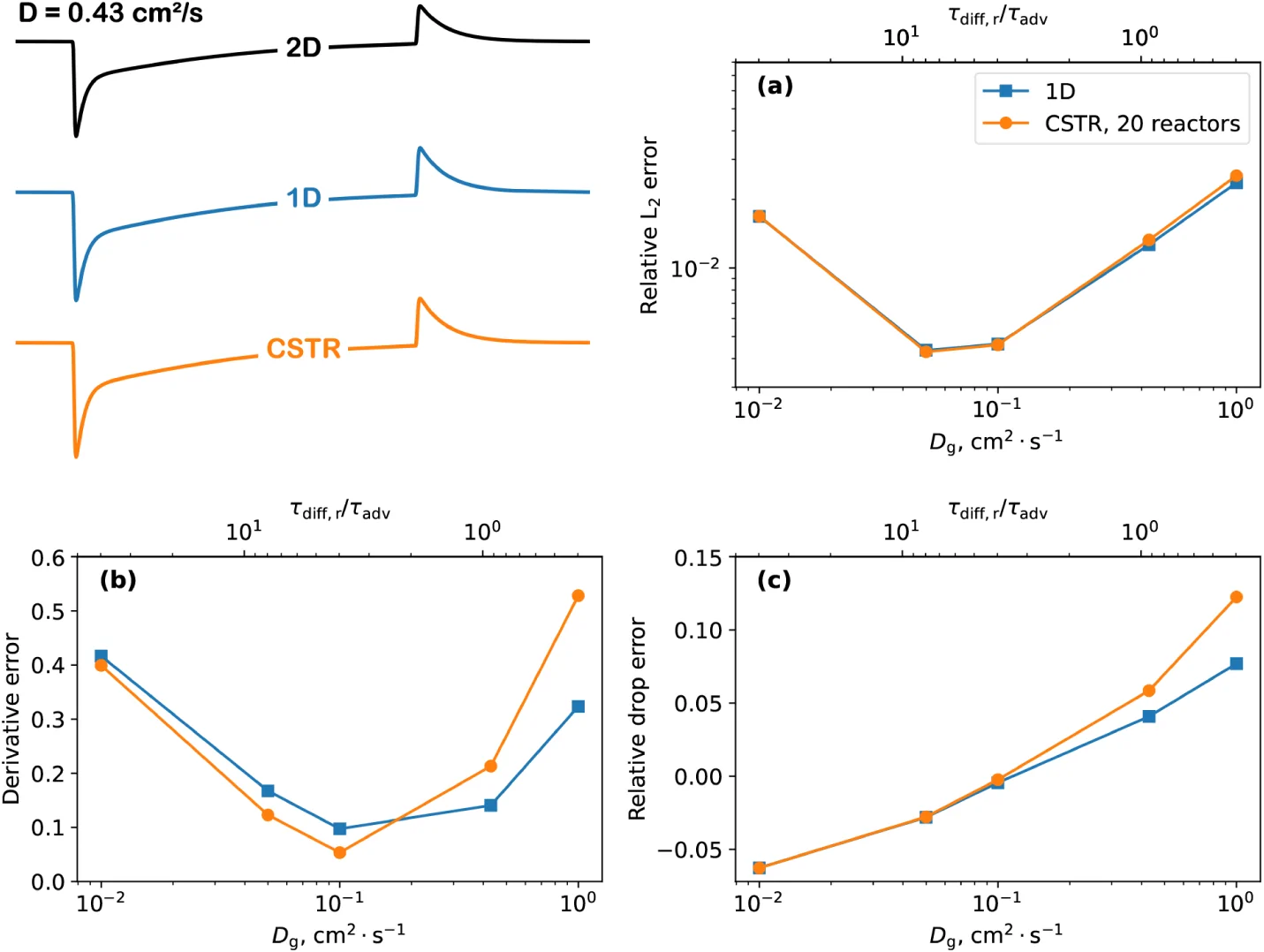
Various metrics of error between the simplified models and the benchmark 2D model at different diffusion coefficients for a fixed flow rate (*F*_st_ = 2.5 cm^3^ s^–1^). Top left: uptake curves generated by the three models at *D*_g,st_ = 0.43 cm^2^ s^–1^, which is a typical diffusion coefficient for conditions of uptake experiments. Under those conditions, predicted uptake curves look almost identical. Plots (a–c) show solution errors of simplified models as a function of diffusion coefficient. (a) Relative *L*_2_ error, (b) derivative error, and (c) relative drop error.

Sensitivities of a simulated uptake curve on a reactive substrate are shown in Figure 7 to illustrate the impact of each parameter on the uptake curve.

**Figure 7:**
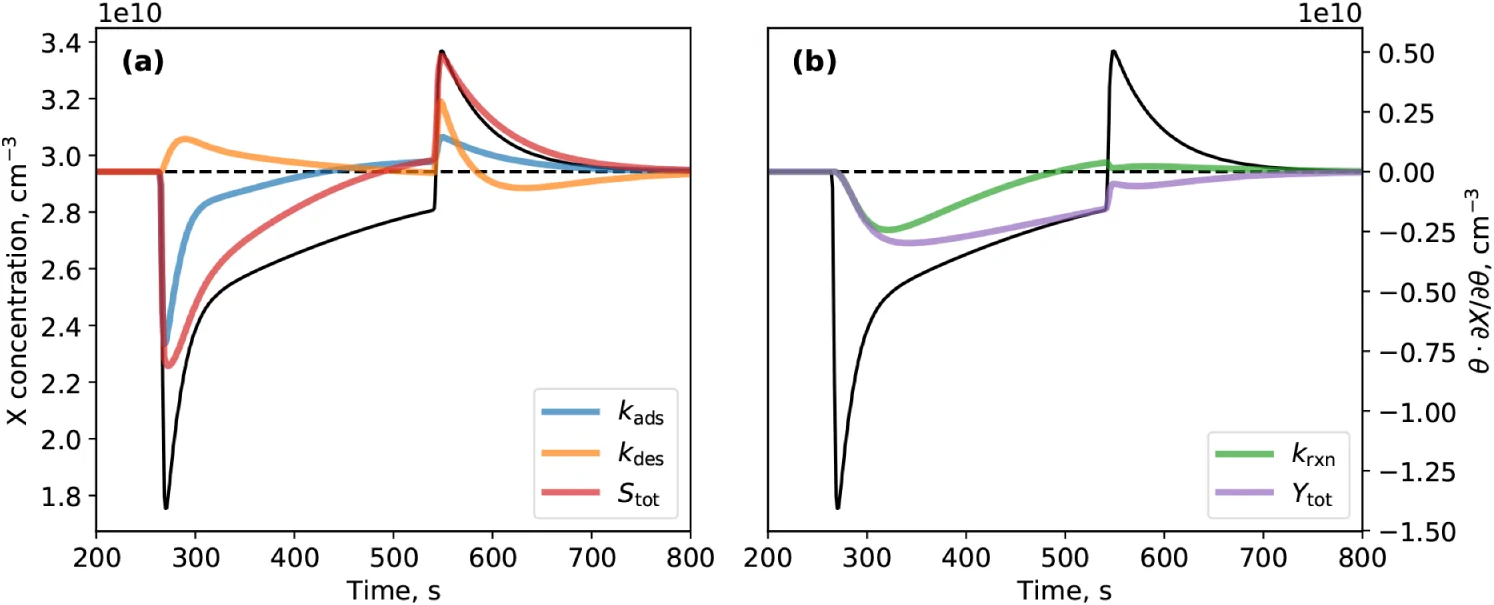
Relative sensitivities of an example uptake curve on a reactive substrate with respect to (a) sorption-related parameters and (b) reaction-related parameters. Solid black curves represent the simulated uptake curve, and the horizontal dashed lines indicate the feed concentration/zero sensitivity. *θ* stands for any mechanism parameter: *k*_ads_, *k*_des_, *k*_rxn_, *S*_tot_, or *Y*_tot_.

Since uptake curves exhibit peak sensitivity to parameters (*k*_rxn_, *Y*_tot_) responsible for chemical reactions only during the later stages of the experiment, one may expect that sampling the system for a longer duration at the quasi-steady state may improve fit accuracy. Accordingly, we investigated the accuracy of fitted (*k*_rxn_, *Y*_tot_) as a function of exposure time, τ_exp_ = *t*_end_ – *t*_start_, by generating 6 uptake curves with τ_exp_ = 100–600 s (Figure 8a). All these curves were produced using identical experimental conditions and kinetic parameters, representative of HgCl_2_ uptake on NaCl, a strongly reactive process (parameters listed in Table 2). When fitting these curves without added noise, the starting kinetic parameters were recovered almost exactly for all τ_exp_. This is expected, as the inverse problem is well-posed because each parameter affects the uptake curve uniquely (Figure 7), and the source of uncertainty in fitted parameters is experimental noise. To further demonstrate that the parameter set describing each uptake curve is unique, we varied each parameter by increasing and decreasing it by a factor of 3 and calculated the corresponding uptake curves. As shown in Figure S1, clearly, each parameter affects the shape of an uptake curve in a unique way. Indeed, varying *k*_ads_ changes mostly the magnitude of the drop at 100 s, *k*_des_ mostly affects the magnitude of the peak at 400 s, *k*_rxn_ changes only the rate of linear recovery between 100 and 400 s, *S*_tot_ affects all three regions very significantly with exponential recovery at 100–400 s, and *Y*_tot_ affects the 100–400 s recovery (but with a different slope than that caused by changing *k*_rxn_) and also to some extent the magnitude of the desorption peak at 400 s.

**Figure 8:**
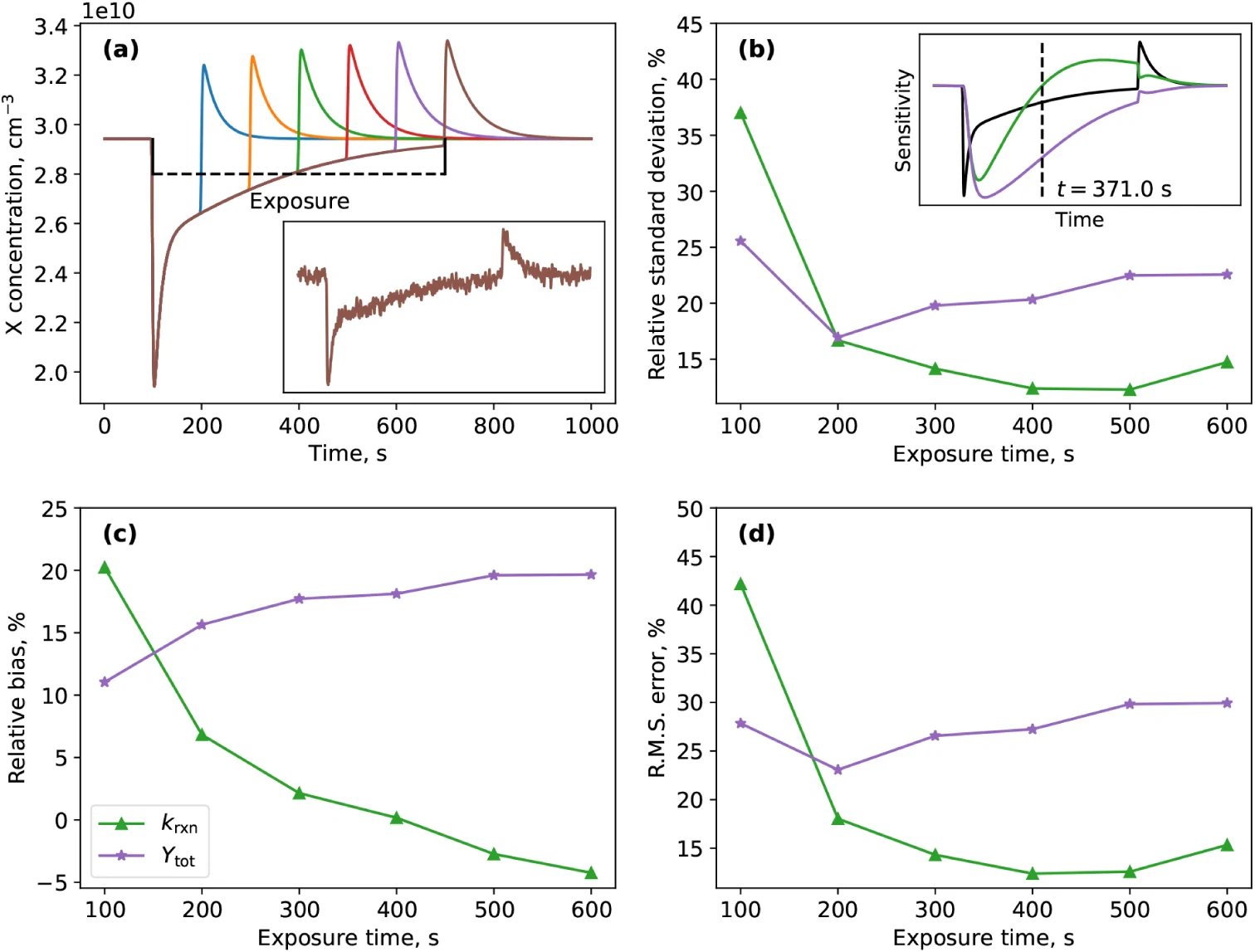
Accuracy of retrieved *k*_rxn_ and *Y*_tot_ parameters as a function of exposure time. Statistics were collected from 100 trials, and data points that converged to a trivial solution (error greater than 90%) were excluded. (a) Uptake curves generated by varying the exposure time (the inset shows one such curve with added artificial noise); (b) relative standard deviation with respect to *k*_rxn_ and *Y*_tot_ (the inset shows sensitivities of the uptake curve with τ_exp_ = 600 s); (c) relative bias; and (d) root-mean-square error of fitted parameters *k*_rxn_ and *Y*_tot_.

**Table 2:** Parameters That Were Extracted from Experimental Uptake Curves Shown in Figure 9. (a) Primary fitted parameters derived from experimental uptakes of HgCl_2_ on NaCl (Figure 9a) and on levoglucosan (Figure 9b). Experimental conditions are described in Table S2. (b) Secondary parameters, calculated from the primary fitted parameters, that can be useful for material characterization or atmospheric modeling

Parameter	NaCl	Levoglucosan	Unit
(a) Fitted parameters			
*k*_ads_	(2.0 ± 0.1) × 10^–12^	(5.7 ± 0.3) × 10^–12^	cm^3^ s^–1^
*k*_des_	(1.77 ± 0.08) × 10^–2^	(3.15 ± 0.05) × 10^–2^	s^–1^
*k*_rxn_	(2.4 ± 0.2) × 10^–16^	(0.0 ± 5.8) × 10^–16^ → 0	cm^2^ s^–1^
*S*_tot_	(3.8 ± 0.1) × 10^13^	(1.19 ± 0.03) × 10^13^	cm^–2^
*Y*_tot_	(8.6 ± 0.2) × 10^13^	(0.0 ± 1.7) × 10^17^ → 0	cm^–2^
(b) Derived parameters			
*K*_ads_	(1.14 ± 0.08) × 10^–10^	(1.80 ± 0.08) × 10^–10^	cm^3^
*K*_rxn_	(1.4 ± 0.1) × 10^–14^	—	cm^2^
*K*_sa,r_	(4.3 ± 0.3) × 10^3^	(2.1 ± 0.1) × 10^3^	cm
*K*_sa,ur_	(2.0 ± 0.2) × 10^3^	—	cm
κ_p_	41 ± 3	—	cm s^–1^
*K*_sa,tot_	(2.5 ± 0.2) × 10^7^ [Table-fn tbl2fn4]	—	cm
*A*_sp_	1.84 × 10^5^ ^b^	2.37 × 10^5^ ^c^	cm^2^ g^–1^
*K*_p,r_	(7.9 ± 0.6) × 10^8^ ^b^	(5.1 ± 0.3) × 10^8^ ^c^	cm^3^ g^–1^
*K*_p,ur_	(3.6 ± 0.3) × 10^8^ ^b^	—	cm^3^ g^–1^
*K*_p,tot_	(4.5 ± 0.3) × 10^12^ ^b^ [Table-fn tbl2fn4]	—	cm^3^ g^–1^
γ_0_	(2.0 ± 0.1) × 10^–2^	(1.76 ± 0.09) × 10^–2^	—
γ_qss,ur_	(1.07 ± 0.08) × 10^–2^	—	—
*C*_ML_	3.2 × 10^14^	2.5 × 10^14^	cm^–2^
χ_S_	(1.17 ± 0.04) ×10^–1^	(4.8 ± 0.1) × 10^–2^	—
χ_Y_	(2.69 ± 0.07) × 10^–1^	—	—
χ_Y_/χ_S_	2.29 ± 0.10	—	—

a Assuming a particle diameter of 150 nm and a material density of 2.17 g cm^–3^.

b Assuming a particle diameter of 150 nm and a material density of 1.69 g cm^–3^.

c Assuming an atmospheric lifetime of particles τ_life_ of 6 × 10^5^ s (1 week).

To challenge the fitting algorithm, 100 random variations of artificial noise were added to each generated uptake curve. Details of how artificial noise was generated are provided in Section S3 of Supporting Information and an example uptake curve with τ_exp_ = 600 s with added artificial noise is shown in the inset of Figure 8a.

Using these noisy curves, three metrics of accuracy were evaluated: relative standard deviation σ̂ (Figure 8b), relative bias b̂ (Figure 8c), and root-mean-square error $E_{\mathrm{RMS}}=\sqrt{{\mathrm{\sigma ̂}}^{2}+{\mathrm{b̂}}^{2}}$ (Figure 8d). The first two parameters quantify random error and systematic bias, whereas the last parameter is a combined metric.

The results show that random noise contributes to both random error and systematic bias in fitted *k*_rxn_ and *Y*_tot_. Random error σ̂ can reach ∼40% for both parameters at shorter exposure times (τ_exp_ = 100 s), but drops to ∼25% as τ_exp_ increases to 200 s. It then further decreases and plateaus for *k*_rxn_, but increases again for *Y*_tot_. The reason for that becomes evident after looking at sensitivities of the uptake curve with respect to *k*_rxn_ and *Y*_tot_ (inset in Figure 8b).

Artificial noise added to generated uptake curves consisted of pure random and cumulative random noise. Pure random noise is an array of normally distributed random numbers and is responsible for high-frequency up and down spikes in the signal. Cumulative random noise is a different array of normally distributed random numbers with a cumulative sum operation applied and is responsible for random drift in the signal. Drift causes the baseline to shift up or down for a duration of the order of 100 s. Such a drift biases *Y*_tot_ in a single direction because ∂*X*/∂ **Y*_tot_* does not change sign over the entire exposure duration. Consequently, the bias accumulates with increasing exposure time, resulting in an error that grows monotonically with exposure duration. Short-to-moderate exposure times are optimal for an accurate retrieval of *Y*_tot_. At extremely short exposure times, random noise can contribute to the error significantly, as sampling is not sufficiently long for random noise to cancel out. At longer exposure times, the bias comes from slow signal drift.

In the case of *k*_rxn_, its sensitivity ∂*X*/∂ **k*_rxn_* switches sign during exposure (at 371 s, as indicated with a dashed line in Figure 8b). Runs with medium to long exposure times continue long enough to include this sign change. As a result, a bias that spans across the sign change will first affect the fitted parameter in one direction and then—in the other, effectively leading to the cancellation of errors. Therefore, moderate-to-long exposure times are recommended for an accurate *k*_rxn_ retrieval. At extremely long exposure times, such as 600 s in the example considered here, the error tends to increase because the area of the positive part of the sensitivity curve starts to exceed the area of the negative part, leading to less efficient cancellation of errors.

## 3 Applications of the Model

In this section, an application of the CSTR model to retrieve kinetic parameters of the Langmuir–Hinshelwood mechanism is showcased using two different uptake curves available from previous experiments in our laboratory.^19,20^ Fitted parameters are scrutinized on the basis of calculated uncertainties to determine if they are meaningful. Recommendations for experimentalists are made on how to reduce the uncertainty in the retrieval of adsorptive and reactive parameters. We discuss additional information that can be learned about the system from fitted parameters and derive constants relevant for atmospheric aerosol modeling. Finally, the use of fitted parameters to make predictions of uptake on aerosol under atmospheric conditions is illustrated.

Examples of uptake curves fitted to two experimental data sets are shown in Figure 9, and the corresponding fitted parameters are listed in Table 2a. Experimental data sets include uptake curves of gaseous HgCl_2_ on solid surfaces of NaCl and levoglucosan, which are a highly reactive substrate and a weakly reactive substrate, respectively. Experimental conditions at which the curves were recorded are provided in Section S4 of the Supporting Information. Prior to fitting, experimental data were preprocessed to correct for baseline signal drift and to determine event times *t*_start_ and *t*_end_. The preprocessing steps are provided in Section S5 of the Supporting Information.

**Figure 9:**
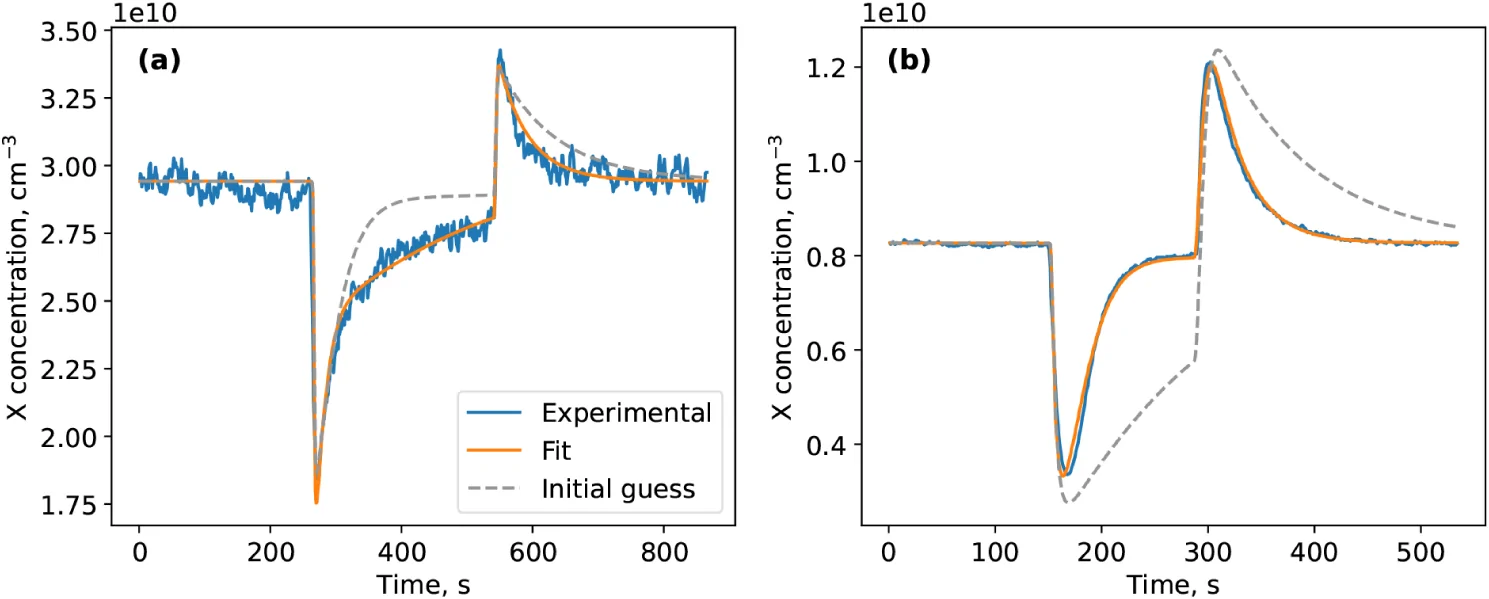
CSTR model fitted to the uptake of gaseous HgCl_2_ on (a) NaCl and (b) levoglucosan. Dashed curves show uptake curves computed using initial guess parameters, while orange curves show uptake curves computed with fitted parameters.

### 3.1 Interpretation of Results and Derivation of Additional Parameters

Kinetic parameters extracted from uptake curves by fitting our model have a physical meaning, and each parameter can provide insights into heterogeneous uptake at the molecular scale. Fitted parameters can also be used to estimate inputs for higher-level models, such as γ, *K*_p_, and *K*_sa_ at different conditions. In this section, the meaning of Langmuir–Hinshelwood parameters is discussed, and several useful constants are derived from them. Derived parameters computed from the example fits are shown in Table 2b.

Because of the slow surface reaction of adsorbed HgCl_2_ on levoglucosan, uncertainties of fitted parameters *k*_rxn_ and *Y*_tot_ are an order of magnitude higher than their nominal values. Therefore, the values of *k*_rxn_ and *Y*_tot_ derived for levoglucosan are meaningless and were rounded off to zero for the purpose of all subsequent calculations.

#### 3.1.1 Initial Uptake Coefficient

The adsorption rate constant *k*_ads_ depends on the initial uptake coefficient of adsorbate molecules colliding with the surface:

29 $$k_{\mathrm{ads}}=\frac{\gamma_{0}\omega }{S_{\mathrm{tot}}}$$

Initial uptake coefficient γ_0_ is generally different from the uptake coefficient γ because the former only includes the adsorption flux, whereas the latter includes the net adsorption/desorption flux. Hence, γ_0_ is equal to γ only at the very start of the experiment, when nothing has been adsorbed yet and there is no desorption or reaction (eq A16). γ_0_ calculated for the adsorption of HgCl_2_ on both NaCl and levoglucosan is approximately 0.02 (Table 2b). Our γ_0_ is comparable in magnitude to the initial uptake coefficient of 0.009 reported for the uptake of NO_2_ on diesel soot.^49^

#### 3.1.2 Nature of Sorptive and Reactive Sites

Surface concentrations of sorptive and reactive sites derived with our model can be related to geometrical and chemical characteristics of the surface. Mao et al.^20^ hypothesized that there are stronger and weaker reactive sites in NaCl, with stronger sites being scarce and corresponding to surface defects of the crystal and with weaker sites being in surplus and corresponding to smooth crystal terraces. They did not provide a hypothesis for the identity of sorptive sites, but assumed that the concentration of sorptive sites was much lower than the concentration of weakly reactive sites. Our model does not resolve multiple kinds of reactive sites, but provides a way to compare the quantities of sorptive and reactive sites, where all possible types of reactive sites are lumped together into an averaged parameter. It is convenient to normalize *S*_tot_ and *Y*_tot_ by monolayer density *C*_ML_, thus yielding monolayer fraction χ_ML_. The smallest unit of a surface that can be used for normalization is a unit cell in the case of a crystalline surface or a molecule in the case of an amorphous surface. If the monolayer density for a cubic crystal with lattice constant *a* is defined as *C*_ML_ = 1/a^2^, then the fraction of adsorptive (χ_S_) or reactive sites χ_Y_ per surface unit cell can be estimated:

30 $$\chi_{S}=\frac{S_{\mathrm{tot}}}{C_{\mathrm{ML}}}\;\mathrm{and}\;\chi_{Y}=\frac{Y_{\mathrm{tot}}}{C_{\mathrm{ML}}}$$

Similarly, monolayer density can be defined in terms of van der Waals diameter of surface atoms or the effective diameter of surface molecules $C_{\mathrm{ML}}=1/\pi {\left(\frac{d}{2}\right)}^{2}$ . Monolayer fraction is the ratio of the number of sites to the number of unit cells/molecules present on the surface and can hint at the identity of sites (whether they are smooth crystal terraces, surface defects, functional groups, etc.).

Monolayer fractions of sorptive sites and reactive sites were found to be 12% and 27%, respectively, for NaCl. With reactive sites taking up nearly one-third of the surface, they could indeed be smooth crystal terraces. Sorptive sites, being more limited in number, are likely to be either surface defects or functional groups. These results support the hypothesis of Mao et al. in a more quantitative manner. χ_Y_ was not calculated for levoglucosan due to it being effectively nonreactive, but χ_S_ was 5%—comparable in magnitude to NaCl.

#### 3.1.3 Partitioning Constant

To enable the simulation of heterogeneous uptake in equilibrium aerosol models, the surface-area-normalized gas-particle partitioning constant *K*_sa_ (eq 7) can be derived from fitted parameters. Depending on what state of the system we want to capture in an equilibrium model, two types of partitioning constants can be defined. A total partitioning constant *K*_sa,tot_ captures the concentrations of both adsorbate *X*_s_ and the product *P*. A reversible partitioning constant only captures the concentration of the adsorbate and can be further split into reacted *K*_sa,r_ and unreacted *K*_sa,ur_ partitioning constants depending on the state of the surface reaction.

Expressions for reversible and total partitioning constants are derived in Appendices A1.1 and A1.2, respectively. The final expressions are

31a $$K_{\mathrm{sa},r}=S_{\mathrm{tot}}K_{\mathrm{ads}}$$

31b $$K_{\mathrm{sa},\mathrm{ur}}=\frac{K_{\mathrm{sa},r}}{1+K_{\mathrm{rxn}}Y_{\mathrm{tot}}}$$

31c $$K_{\mathrm{sa},\mathrm{tot}}=K_{\mathrm{sa},\mathrm{ur}}+\underset{\kappa_{p}}{\underset{︸}{\frac{Y_{\mathrm{tot}}K_{\mathrm{ads}}k_{\mathrm{rxn}}S_{\mathrm{tot}}}{1+K_{\mathrm{rxn}}Y_{\mathrm{tot}}}}}\tau_{\mathrm{life}}$$

where *K*_ads_ is the Langmuir equilibrium constant given by *K*_ads_ = *k*_ads_/*k*_des_, *K*_rxn_ is a shorthand constant (not an equilibrium constant) employed for brevity and defined as *K*_rxn_ = *k*_rxn_/*k*_des_, and τ_life_ is the characteristic atmospheric lifetime of particles. The coefficient κ_p_ represents the first-order rate of uptake through surface reaction normalized by gas-phase concentration. When multiplied by the time the particle spent in the atmosphere and by the gas-phase concentration, it approximately gives the surface concentration of the product, *P ≈ κ*_p_τ_life_ *X*_gs_.

In case an atmospheric model can only represent phase transfer on a per-mass basis (not per-area), surface-normalized partitioning constants *K*_sa_ can be converted to conventional (mass-basis) partitioning constants *K*_p_,^28,63^

32 $$K_{p}=K_{\mathrm{sa}}A_{\mathrm{sp}}$$

where *A*_sp_ is the specific area of aerosol, which is defined as the ratio of the surface area of a particle to its mass. For a solid spherical particle, it is given by

33 $$A_{\mathrm{sp}}=\frac{3}{R_{p}\rho }$$

where *R*_p_ is the radius of the particle and ρ is its material density.

*K*_sa_ values are size-independent and are the natural parameters for describing surface processes. *K*_p_ values require making an assumption about particle size and material density and thus are only applicable to a narrow size range of particles. Nevertheless, we report some example *K*_p_ values in Table 2 for an assumed particle size.

#### 3.1.4 Initial and Time-Dependent Uptake Coefficients

Uptake curves simulated by the model can be converted to the uptake coefficient form by calculating the ratio of the net uptake flux by the surface *r*_uptake_ to the total thermal collision flux of X with the surface:

34a $$\gamma \left(t\right)=\frac{r_{\mathrm{uptake}}\left(t\right)}{X_{\mathrm{gs}}\omega }$$

34b $$r_{\mathrm{uptake}}\left(t\right)=k_{\mathrm{ads}}f_{\mathrm{sw}}\left(t\right)X_{\mathrm{gs}}\left(S_{\mathrm{tot}}-X_{s}\right)-k_{\mathrm{des}}X_{s}$$

The conversion requires the knowledge of the time evolution of states *X*_gs_ and *X*_s_, which are known from the model upon simulation of an uptake curve, but are not measured in an experiment. Thus, another approach has traditionally been used by experimentalists to convert uptake curves to the uptake coefficient form. The traditional approach involves calculating the observed first-order loss rate constant *k*_obs_ and correcting for diffusion through the resistance model (eqs 5 and 11), which yields an effective first-order uptake rate constant *k*_uptake_ = (2/*R*)­(*r*_uptake_/*X*_gs_) (in per-volume, not per-surface units):^20^

35a $$k_{\mathrm{obs}}\left(t\right)\approx \frac{F}{\pi R^{2}L}\ln\frac{X_{\mathrm{feed}}}{X\left(t\right)}$$

35b $$\frac{1}{k_{\mathrm{uptake}}\left(t\right)}\approx \frac{1}{k_{\mathrm{obs}}\left(t\right)}-\frac{1}{k_{\mathrm{diff}}}$$

35c $$\gamma \left(t\right)=\frac{R}{2}\frac{k_{\mathrm{uptake}}\left(t\right)}{\omega }$$

This approach involves several assumptions; eq 47 assumes that *k*_obs_ is constant for a parcel of gas as it travels through the entire length of the reactor, which may not be true at the start of uptake when the concentration drop is large; eq 48 assumes that diffusion is at steady state, which may also break at the very start of uptake. We compare uptake coefficients estimated using this approach with uptake coefficients computed using our model.

Experimental studies typically evaluate and report two constants—an initial uptake coefficient, which is determined at the very start of the experiment, and a final uptake coefficient, which is determined at the time just before desorption starts. Here, we refer to the uptake coefficient calculated from an uptake curve at the start of uptake as the “apparent initial uptake coefficient” because it is not necessarily equal to the true uptake coefficient on a pure surface and denote it as γ_0,app_. The final uptake coefficient is denoted as γ_fin_.

We present in Figure 10 uptake coefficient curves for NaCl and levoglucosan calculated directly with the model from their fits (eq 45). We also calculated γ_0,app_ and γ_fin_ using the first-order observed rate approximation (eq 47). Approximated uptake coefficient values are compared to uptake coefficients calculated directly by the model in Table 3. The true (eq 45) initial uptake coefficient for a pure surface was calculated from fitted parameters and is provided for comparison.

**Table 3:** Apparent Initial Uptake Coefficient and Final Uptake Coefficient Calculated in Two Ways: Exactly from the Definition of Uptake Coefficient (eq 45) and Using the Observed First-Order Rate Constant Approximation (eq 47)

Parameter	Exact (eq 45)	Approximation (eq 47)	% diff.
(a) NaCl uptake coefficient estimates			
γ_0,app_	1.58 × 10^–2^	1.53 × 10^–2^	–3%
γ_fin_	1.37 × 10^–3^	1.27 × 10^–3^	–7%
γ_0_	1.98 × 10^–2^	—	—
(b) levoglucosan uptake coefficient estimates			
γ_0,app_	1.05 × 10^–2^	0.997 × 10^–2^	–5%
γ_fin_	4.24 × 10^–4^	3.95 × 10^–4^	–7%
γ_0_	1.76 × 10^–2^	—	—

**Figure 10:**
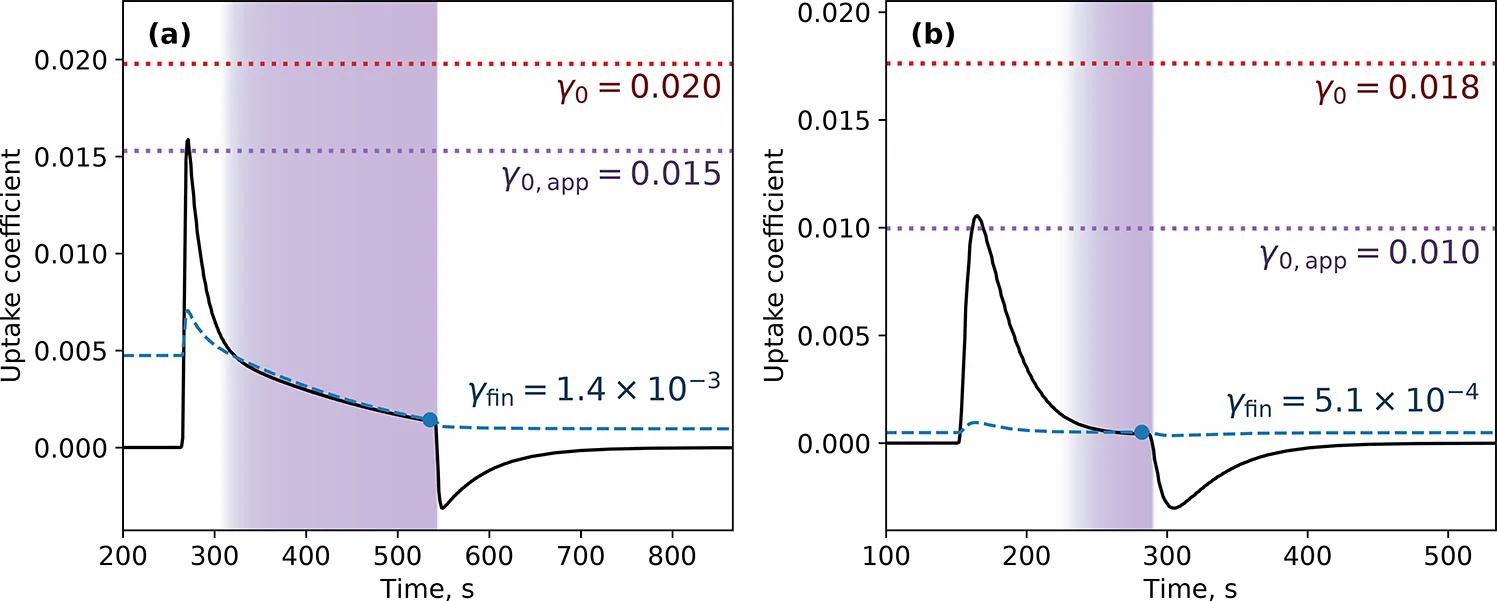
Uptake coefficients obtained from (a) NaCl and (b) levoglucosan fits shown with black lines. Shaded regions show where *dX*_s_/*dt* ≈ 0 and sorption is at steady state—the region of validity of the quasi-steady-state approximation. Dotted horizontal lines show initial uptake coefficients calculated with eqs 75 and 47. Blue dashed curves show time-dependent uptake coefficients calculated with the quasi-steady-state approximation (eq A19).

Fitted parameters obtained with our model can be used to extrapolate the initial uptake coefficient, γ_0_, which cannot be measured experimentally due to its limiting nature. As seen in Figure 10, the true γ_0_ is always higher than the maximum uptake coefficient observed in an experiment, γ_0,app_. This is because the rate of uptake is the fastest when none of the sorptive sites on the surface are occupied. Sorptive peaks get smoothed in experiments because when an experiment begins, the injector tube needs to be retracted (Figure 1), which is not instantaneous. While the injector is being moved, the part of the surface near the outlet of the reactor starts getting covered in adsorbate, while the part near the inlet is still pure. As a result, when the injector is fully retracted and the maximum drop in concentration is observed, a fraction of the surface near the outlet is already partially deactivated, and the maximum theoretical uptake rate can never be observed. This effect is captured in the uptake model as well through the switching function, *f*_sw_(*t*), which also smooths the sorptive peaks and causes the apparent initial uptake coefficient to be underpredicted. Another parameter that can be useful for kinetic aerosol models is the unreacted quasi-steady-state uptake coefficient, γ_qss,ur_. This parameter characterizes uptake by a surface at a time when sorption has reached steady state already (*dX*_*s*_/*dt* ≈ 0), but the surface reaction is still in its initial stage (*P* ≈ 0). With the exception of the initial uptake coefficient, which is not dependent on gas-phase concentration, uptake coefficients are not constant with respect to *X*_gs_, and γ_qss,ur_ was derived through an extrapolation to low concentrations, where the uptake coefficient can be approximated as a constant. Thus, due to the underlying assumptions, γ_qss,ur_ is applicable to the modeling of aerosols where reversible sorption and irreversible reaction occur at vastly different time scales and can be decoupled, but it is not relevant for uptake experiments where sorption and surface reaction overlap due to high concentrations.

Detailed derivations for γ_0_ and γ_qss,ur_ in terms of fitted parameters are provided in Appendix A2, while the final expressions are listed here. Applications of these parameters for the purpose of aerosol modeling are described in the subsequent section.

36a $$\gamma_{0}=\frac{k_{\mathrm{ads}}S_{\mathrm{tot}}}{\omega }$$

36b $$\gamma_{\mathrm{qss},\mathrm{ur}}=\gamma_{0}\frac{K_{\mathrm{rxn}}Y_{\mathrm{tot}}}{1+K_{\mathrm{rxn}}Y_{\mathrm{tot}}}$$

### 3.2 Modeling of Heterogeneous Uptake on Aerosol Particles in the Atmosphere

One of the main applications of extracted Langmuir–Hinshelwood parameters is to model heterogeneous uptake on aerosol particles. The most accurate way to represent such a process is to solve the system of differential eqs 37a–d, which captures the transport of reactant X through diffusion within the parcel of air and its loss to aerosol particles. Heterogeneous chemistry is represented here explicitly through the two steps of the Langmuir–Hinshelwood mechanism:

37a $$\frac{dX}{dt}=-k_{\mathrm{diff}}\left(X-X_{\mathrm{gs}}\right)+Ġ-\mathrm{L̇}$$

37b $$\frac{dX_{\mathrm{gs}}}{dt}=k_{\mathrm{diff}}\left(X-X_{\mathrm{gs}}\right)+A_{v}\left[k_{\mathrm{des}}X_{s}-k_{\mathrm{ads}}X_{\mathrm{gs}}\left(S_{\mathrm{tot}}-X_{s}\right)\right]$$

37c $$\frac{dX_{s}}{dt}=k_{\mathrm{ads}}X_{\mathrm{gs}}\left(S_{\mathrm{tot}}-X_{s}\right)-k_{\mathrm{des}}X_{s}-k_{\mathrm{rxn}}X_{s}\left(Y_{\mathrm{tot}}-P\right)$$

37d $$\frac{dP}{dt}=k_{\mathrm{rxn}}X_{s}\left(Y_{\mathrm{tot}}-P\right)$$

where the terms Ġ and L̇ represent the rates of gas-phase generation and loss of compound X, and *k*_diff_ is a first-order rate constant that describes the transport of X by diffusion from bulk air to the proximity of the particle surface. For monodisperse particles of radius *R*_p_, *k*_diff_ is given by^64,65^

38 $$k_{\mathrm{diff}}=4\pi R_{p}D_{g}N_{p}$$

where *N*_p_ is the particle number density. *A*_v_ in eq 53 is the conversion factor between surface and volumetric concentrations. In the case of uptake on aerosol particles, *A*_v_ is the surface area of particles per unit volume:

39 $$A_{v}=4\pi R_{p}^{2}N_{p}$$

Transition correction is not taken into account in this case because we apply the model to large particles in examples discussed in the paper, with the goal of obtaining order-of-magnitude estimates of uptake on aerosol under atmospheric conditions. Quantitative predictions would require a rigorous treatment of aerosol particle size distributions and compositions, emission and removal rates, gas-phase reactant production, etc.

Using parameters for the Langmuir–Hinshelwood mechanism derived from laboratory experiments involving the uptake of HgCl_2_ on NaCl and levoglucosan surfaces in a flow reactor, we simulated the uptake of HgCl_2_ on aerosol particles under atmospheric conditions. The concentration of X was assumed to be constant for the purpose of this illustrative run, and thus, eq 52 was ignored. Otherwise, the generation and loss terms Ġ and L̇, which capture gas-phase atmospheric processes, would also need to be defined. The parameters used in this illustrative modeling run are listed in Table 4.

**Table 4:** Parameters Used in the Illustrative Simulation of Uptake on Aerosol Particles

Parameter	Value	Unit
*N*_p_	1000	cm^–3^
*R*_p_	200	nm
*D*_g_	0.1^a^	cm^2^ s^–1^
*X*	100 (2.01 × 10^5^)^b^	pg m^–3^ (cm^–3^)
*M*_w_	271.52	g mol^–1^
*T*	300^c^	K

a Diffusion coefficient of HgCl_2_ in air at atmospheric pressure.

b A higher HgCl_2_ concentration was used to validate the low-concentration approximations made in the derivation of partitioning constants and uptake coefficients.

c Only used for uptake coefficient calculation in postprocessing.

Results of the uptake simulations for aerosol particles are shown in Figure 11a. Gas-phase concentration near the particles, *X*_gs_, rapidly drops after uptake begins and, for nonreactive particles (levoglucosan), recovers within 10^2^–10^3^ seconds. The drop in concentration is under 1% because under these conditions gas-phase diffusion is faster than uptake, keeping *X*_gs_ nearly constant. The rapid initial uptake is driven by reversible sorption. After sorption reaches steady-state, uptake becomes reaction-driven or stops in the case of nonreactive particles. Reactive uptake maintains a constant gas-phase concentration *X*_gs_ slightly below ambient concentration *X* until the reactive sites start to run out after about 10^6^ seconds of uptake. As all of the reactive sites get used up at around 10^7^–10^8^ seconds, uptake stops, and the gas-phase concentration near the particles *X*_gs_ returns to the ambient concentration, *X*. In the absence of uptake, gas-phase concentration near the particles is fully replenished by diffusion from the bulk of the gas phase.

**Figure 11:**
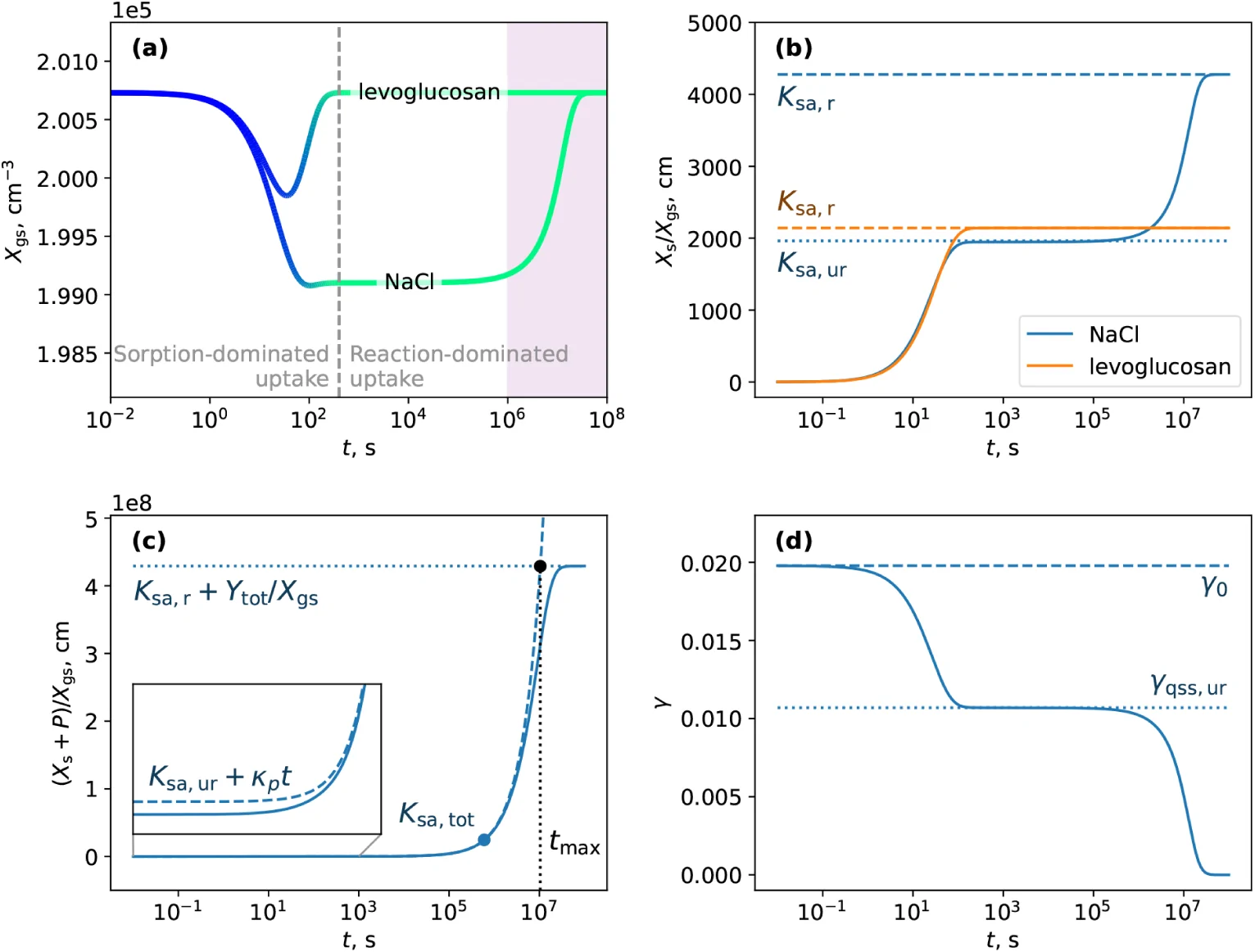
Results of a simulation of the uptake of HgCl_2_ on aerosol particles. (a) Gas-phase concentration of the reactant near the surface of a particle as a function of time. Color indicates the magnitude of *dX*_s_/*dt*—green when the derivative is close to zero and blue otherwise. (b) The ratio of surface concentration of adsorbate *X*_s_ to gas-phase concentration *X*_gs_, along with reversible gas-particle partitioning constants *K*_sa,r_ and *K*_sa,ur_ shown with horizontal lines. (c) The ratio of the total particle-phase concentration *X*_s_ + *P* to gas-phase concentration *X*_gs_, along with the time-dependent total gas-particle partitioning constant shown with a dashed curve. The maximum possible particle-phase concentration is shown with a dotted curve. *K*_sa,tot_ evaluated at an atmospheric lifetime of 1 week is shown with a marker. (d) Time-dependent uptake coefficient computed directly from simulated *X*_s_ and *X*_gs_, along with γ_0_ and γ_qss,ur_ shown with horizontal lines.

The ratios of aerosol-phase to gas-phase concentrations and uptake coefficients computed in numerical simulations under atmospherically relevant conditions can be compared against partitioning constants and uptake coefficients derived under low-concentration assumption in the previous section. Figure 11b compares the computed ratio *X*_s_/*X*_gs_ with the reversible partitioning constants *K*_sa,r_ and *K*_sa,ur_. In the case of nonreactive particles (levoglucosan), *X*_s_/*X*_gs_ converges to the reacted reversible partitioning constant *K*_sa,r_ (eq 40) at the time when sorption has reached equilibrium (around 10^2^ s). In the case of reactive particles (NaCl), *X*_s_/*X*_gs_ plateaus twice, converging to the unreacted reversible partitioning constant *K*_sa,ur_ (eq 41) at the first plateau and to the reacted reversible partitioning constant *K*_sa,r_ (eq 40) at the second plateau. The first plateau corresponds to the quasi-steady state when sorption is at equilibrium, but the surface reaction is still ongoing at approximately a constant rate. The rate of adsorbate *X*_s_ gain through adsorption is equal to the rate of adsorbate loss through desorption and the surface reaction. Thus, a constant *X*_s_, which is below the Langmuir equilibrium value, was maintained. Once the reactive sites begin to run out, the reaction slows, causing a spike in *X*_s_ and leading to the second plateau. At the second plateau, the reaction is no longer consuming adsorbate, and *X*_s_ is at Langmuir equilibrium.

Figure 11c compares (*X*_s_ + *P*)/*X*_gs_, shown with a solid blue line, against the time-dependent total partitioning constant *K*_sa,tot_ (eq 42), shown with a dashed blue line. The latter equation assumes that sorption reaches quasi-steady state instantly, which leads to a slight overprediction of the particle-bound mercury concentration at the start of uptake, but the curves converge around 10^2^ seconds. Driven by the surface reaction, the particle-phase concentration increases linearly (when plotted on a linear time scale) from there until the reactive sites begin to run out. At that point, the particle-phase concentration converges to a constant value of *K*_sa, r_ *X*_gs_ + *Y*_tot_, while eq 42 breaks by continuing to predict a linear increase. The time when the particle phase concentration predicted by eq 42 exceeds the maximum possible particle phase is τ_max_ (eq 73), which is around 10^7^ seconds in this case—far beyond the typical atmospheric lifetime of particles.

Figure 11d compares the time-dependent uptake coefficient calculated with eq 45 (without the switching function; *f*_sw_(*t*) = 1) with the initial uptake coefficient γ_0_ (eq 50) and the quasi-steady-state unreacted uptake coefficient γ_qss,ur_ (eq 51). The two regimes of uptake are clearly distinct in this figure. The process starts at γ_0_ with fast sorption-dominated uptake. It drops by almost a factor of 2 and plateaus at γ_qss,ur_ after approximately 10^2^ seconds. The uptake coefficient is then constant, while the surface engages in slower reaction-dominated uptake until it drops to zero when the reaction stops around 10^6^–10^7^ seconds. The drop of γ to zero happens beyond the typical atmospheric lifetime of a particle, and sorption-dominated uptake only lasts for minutes at the start of the process. Therefore, it is reasonable to approximate the uptake coefficient with a constant γ_qss,ur_ in a kinetic aerosol model, while the sorption-dominated portion can be captured in an equilibrium manner using *K*_sa,ur_.

While a kinetic model that numerically solves the Langmuir–Hinshelwood mechanism provides the most detailed information about an aerosol system and results in the highest accuracy, accurate predictions at a broad range of conditions can be made analytically using the time-dependent partitioning constant, *K*_sa,tot_. It is derived from parameters of the Langmuir–Hinshelwood mechanism and relates the gas-phase concentration to the particle-phase concentration (eq 42). For instance, *K*_sa,tot_ can predict the quantities relevant for field and modeling atmospheric mercury studies, which usually do not resolve speciation of particle-bound mercury (whether it is *X*_s_ or *P*), lumping these forms together.^100^ What matters is the relationship between gaseous oxidized mercury (GOM) concentration, *C*_GOM_, and the particle-bound mercury (PBM) concentration, *C*_PBM_. *C*_GOM_ is directly related to *X* used in this study, except that it is typically expressed as a mass-based concentration with units of pg m^–3^ rather than as a number concentration. *C*_PBM_ is the concentration of the particle phase mercury per unit volume of air, also typically expressed in units of pg m^–3^, unlike *X*_s_ + *P*, which is the concentration per unit surface area of a particle. *A*_v_ can be used as a conversion factor between per-surface area of a particle and per-volume of air representations (eq 57). Time-dependent *K*_sa,tot_ can relate gas-phase concentration to particle-phase concentration for both representations of concentration:

40a $$X_{s}+P=\left(K_{\mathrm{sa},\mathrm{ur}}+\kappa_{P}t\right)X_{\mathrm{gs}}$$

40b $$C_{\mathrm{PBM}}=\left(K_{\mathrm{sa},\mathrm{ur}}+\kappa_{P}t\right)A_{v}C_{\mathrm{GOM}}$$

Equations 58 and 59 are a simple model that involves several assumptions, which restrict the range of conditions when the model can be applied. Particle lifetime must be greater than the time required to reach quasi-steady state τ_qss_ (eq 84) and less than the time when the surface reaction reaches completion τ_max_ (eq 73). Gas-phase concentration *X*_gs_ must be sufficiently low so that reversible sorption and surface reaction can be decoupled and that the first-order approximation is applicable. For a gas-phase concentration *C*_GOM_ of 100 pg m^–3^, which is equivalent to *X*_gs_ of 2.01 × 10^5^ cm^–3^ and is considered high, the linear approximation held up well (Figure 11c). For reference, field measurements report the sum of *C*_GOM_ and *C*_PBM_ ranging from 18 to 160 pg m^–3^.^66–68^ The time bounds τ_qss_ and τ_max_ for a NaCl particle at this gas-phase concentration were 26 s and 1.1 × 10^7^ s, respectively. To illustrate the model, Figure 12 shows *C*_PBM_ computed for a range of *C*_GOM_ (up to 100 pg m^–3^) and a range of particle residence times for levoglucosan and NaCl particles. *A*_v_ was calculated assuming the same parameters as given in Table 4. Since levoglucosan is considered nonreactive, κ_P_ = 0, and eq 59 predicts no time dependence. PBM concentrations are only shown for this particle type at one time point—the time when sorption on levoglucosan reaches steady state τ_qss_ = 32 s. For reactive NaCl, PBM concentrations were evaluated at a broad range of times, starting from the time when sorption is equilibrated and up to the maximum expected atmospheric lifetime of a particle. This approach can be used to solve the inverse problem as well—given PBM concentrations from atmospheric measurements, it can calculate the associated GOM concentrations.

**Figure 12:**
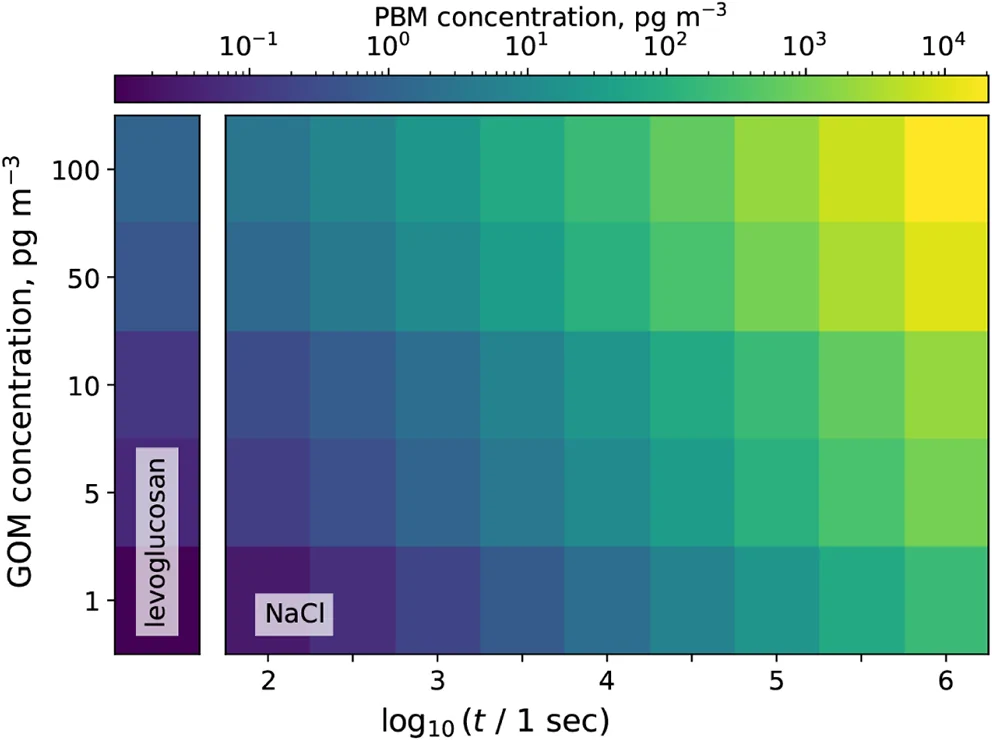
A heatmap of PBM concentrations as a function of GOM concentration (vertical axis) and particle residence time (horizontal axis) computed for levoglucosan (left) and NaCl (right).

## 4 Conclusions

In this work, we developed a framework for analyzing experimental heterogeneous uptake curves obtained in flow reactors. The framework is implemented as a numerical model that is able to deconvolute reversible sorption from surface reactions and separate them from mass transport. Unlike previous kinetic modeling studies,^44–46^ our model features automated fitting and includes surface reactions to derive the fundamental kinetic parameters of the mechanism.

To ensure physical accuracy, model development was performed in steps, starting from a rigorous 2D implementation and then simplifying it to a 1D model and subsequently to a chained CSTR model. Benchmarking the simplified models against the 2D model under relevant conditions demonstrated their performance with comparable accuracy but significantly faster speed. The chained CSTR model was implemented in a custom code and made open-source. A user-friendly web application providing a convenient graphical interface was also published. The fast performance of the chained CSTR model allows it to be run not only in a predictive mode, where an uptake curve is simulated from user-provided parameters, but also in a fitting mode, where an inverse problem is solved by fitting the model to experimental data to obtain the parameters of the mechanism. Our implementation of the CSTR model is able to retrieve reaction parameters within the Langmuir–Hinshelwood mechanism. Other mechanisms can be implemented in a straightforward manner.

Analysis of experimental data using our model can help design optimal experiments. The model can guide the user if certain experimental conditions need to be adjusted to enhance the sensitivity of the uptake curves to certain model parameters. A possible experimental workflow includes running a preliminary experiment, fitting our model to the experimental uptake curve, and using the sensitivity information to optimize the experimental parameters, including the gas pressure, flow velocity, and total uptake duration, for subsequent runs.

Simulations of heterogeneous uptake on aerosol particles, using kinetic parameters obtained with our model, suggested that at atmospheric trace gas concentrations, which are commonly many orders of magnitude lower than those in laboratory experiments, uptake occurs in two steps, which can be decoupled and treated separately. Uptake through reversible sorption is fast and reaches a steady state within seconds to minutes after aerosol particles enter the atmosphere, while uptake through surface reaction is slower and can take days to weeks to reach completion. Decoupling these two surface processes allowed us to derive several analytical equations that can be used to analyze system behavior and to represent heterogeneous reactions in atmospheric models. Simulations have also shown that the amount of reactant present in the particle phase has a linear dependence on gas-phase concentration under atmospheric conditions. Meanwhile, the dependence is nonlinear at high concentrations that are used in uptake experiments. Taking advantage of the ability to decouple sorption and surface reaction, we have derived a set of partitioning constants and uptake coefficients that can be calculated directly from fitted parameters and then used in aerosol models to simulate heterogeneous uptake on particles under different conditions. Calculating partitioning constants and uptake coefficients from fitted parameters rather than directly from experimental data is the mathematically correct way to extrapolate from a nonlinear regime to a linear regime.

Finally, based on the conclusions drawn from aerosol uptake simulations, a simple analytical model was derived that can predict particle-phase concentration as a function of gas-phase concentration of a reactive component at a broad range of times and concentrations. To illustrate its use, the model was applied to establish a relationship between the concentrations of particle-bound mercury and gaseous oxidized mercury. This relationship can be used to replace the simplest partitioning schemes in aerosol models, where all oxidized mercury produced from gaseous elemental mercury is instantaneously split between GOM and PBM in a certain ratio (typically 1:1).^69^

## Supplementary Material



## Appendix A

### A1 Derivation of Parameters for Equilibrium Aerosol Models

#### A1.1 Reversible Uptake Only

In this section, we derive partitioning constants that only take into account adsorbate present on the surface and not the reaction product:

A1
$$K_{\mathrm{sa}}=\frac{X_{s}}{X_{\mathrm{gs}}}$$

Reversible uptake through sorption does reach an equilibrium and is straightforward to represent in an equilibrium model. Reversible sorption is at equilibrium when *dX*_s_/*dt* ≈ 0. Let us call the state when sorption is at equilibrium the quasi-steady state. It is not necessarily the true steady state because in the true steady state, the surface reaction has gone to completion. But in the quasi-steady state, the reaction may or may not still be ongoing. An analytical expression for *X*_s_ at quasi-steady state can be derived from the rate law for *X*_s_:

A2
$$\frac{dX_{s}}{dt}=k_{\mathrm{ads}}X_{\mathrm{gs}}\left(S_{\mathrm{tot}}-X_{s}\right)-k_{\mathrm{des}}X_{s}-k_{\mathrm{rxn}}X_{s}\left(Y_{\mathrm{tot}}-P\right)=0$$

Let *K*_ads_ = *k*_ads_/*k*_des_ and *K*_rxn_ = *k*_rxn_/*k*_des_; then,

A3
$$X_{s,\mathrm{qss}}=S_{\mathrm{tot}}\frac{K_{\mathrm{ads}}X_{\mathrm{gs}}}{1+K_{\mathrm{ads}}X_{\mathrm{gs}}+K_{\mathrm{rxn}}\left(Y_{\mathrm{tot}}-P\right)}$$

The amount adsorbed at quasi-steady-state, *X*_s,qss_, does not depend linearly on the gas-phase concentration, *X*_gs_, because the number of available sorptive sites is limited. But at low concentrations, like in the atmosphere, the response of *X*_s_ to *X*_g__s_ is approximately linear, and *K*_sa_ is the proportionality constant. We can derive the expression for *K*_sa_ in terms of Langmuir–Hinshelwood parameters by linearizing the function *X*_s,qss_(*X*_gs_). Linearization is done by performing a Taylor expansion around *X*_gs_ = 0 and keeping only the first-order term. Thus, an expression for *X*_s,qss_ in the low coverage limit is obtained:

A4
$$X_{s,\mathrm{qss}}\approx S_{\mathrm{tot}}\frac{K_{\mathrm{ads}}X_{\mathrm{gs}}}{1+K_{\mathrm{rxn}}\left(Y_{\mathrm{tot}}-P\right)}$$

Now, two proportionality constants can be derived from the low coverage limit of *X*_s,qss_. One is *K*_sa,r_, which gives the amount adsorbed at a point in time when the surface reaction has gone to completion (*P* = *Y*_tot_) or when there was no reaction to begin with (*K*_rxn_ = 0):

A5
$$K_{\mathrm{sa},r}=S_{\mathrm{tot}}K_{\mathrm{ads}}$$

Another proportionality constant is *K*_sa,ur_, which gives the amount adsorbed at a point in time when sorption has equilibrated, but the surface reaction has just begun (*P* = 0):

A6
$$K_{\mathrm{sa},\mathrm{ur}}=\frac{K_{\mathrm{sa},r}}{1+K_{\mathrm{rxn}}Y_{\mathrm{tot}}}$$

#### A1.2 Reversible and Reactive Uptake

In this section, we derive a partitioning constant that takes into account both the adsorbate present on the surface and the reaction product. *X*_s_ and *P* correspond to different chemical species, but can be lumped into a single “aerosol phase”:

A7
$$K_{\mathrm{sa},\mathrm{tot}}=\frac{X_{s}+P}{X_{\mathrm{gs}}}$$

Unlike reversible sorption, surface reaction typically proceeds to completion and only reaches equilibrium when all reactive sites have been consumed. In the long time limit, the concentration of formed product *P* is equal to the concentration of reactive sites *Y*_tot_ regardless of the gas-phase concentration *X*_gs_. However, that equilibrium is often unattainable within the lifetime of aerosol particles in the atmosphere due to the slow rate of surface reaction at low Langmuir coverage. For the majority of the lifetime of a particle at atmospheric conditions, the concentration of product can be approximated as proportional to *X*_gs_ and *t*. To derive the proportionality constant, let us start with the rate law for product:

A8
$$\frac{dP}{dt}=k_{\mathrm{rxn}}X_{s}\left(Y_{\mathrm{tot}}-P\right)$$

Under atmospheric conditions, sorption reaches equilibrium within minutes, which is approximately instant relative to the time scale of a surface reaction, which can take weeks to proceed to completion (shown in Section 3.2). Therefore, we can approximate *X*_s_ with our previously derived quasi-steady-state expression *X*_s, qss_ (eq 62):

A9
$$\frac{dP}{dt}\approx k_{\mathrm{rxn}}\left(Y_{\mathrm{tot}}-P\right)S_{\mathrm{tot}}\frac{K_{\mathrm{ads}}X_{\mathrm{gs}}}{1+K_{\mathrm{ads}}X_{\mathrm{gs}}+K_{\mathrm{rxn}}\left(Y_{\mathrm{tot}}-P\right)}$$

Equation A9 can be integrated analytically to yield the dependence of *P* on time. Due to the fast equilibration time of *X*_s_, we can assume that no meaningful amount of product has formed when sorption has reached steady state, thus using the initial condition of 0 for *P*. The solution is then as follows:

A10
$$P\left(t\right)\approx Y_{\mathrm{tot}}-\frac{1+K_{\mathrm{ads}}X_{\mathrm{gs}}}{K_{\mathrm{rxn}}}W_{0}\left(\frac{K_{\mathrm{rxn}}Y_{\mathrm{tot}}}{1+K_{\mathrm{ads}}X_{\mathrm{gs}}}\exp\left(\frac{K_{\mathrm{rxn}}Y_{\mathrm{tot}}-k_{\mathrm{rxn}}S_{\mathrm{tot}}K_{\mathrm{ads}}X_{\mathrm{gs}}t}{1+K_{\mathrm{ads}}X_{\mathrm{gs}}}\right)\right)$$

where *W*_0_(..) is the principal branch of the Lambert W function; eq 69 can be linearized by performing a Taylor expansion with respect to *X*_gs_ and keeping the first-order term:

A11
$$P\left(t\right)\approx \frac{Y_{\mathrm{tot}}K_{\mathrm{ads}}k_{\mathrm{rxn}}S_{\mathrm{tot}}t}{1+K_{\mathrm{rxn}}Y_{\mathrm{tot}}}X_{\mathrm{gs}}$$

The total partitioning constant (eq A7) at a time equal to the lifetime of the particle τ_life_ is given by

A12
$$K_{\mathrm{sa},\mathrm{tot}}=K_{\mathrm{sa},\mathrm{ur}}+\kappa_{p}\tau_{\mathrm{life}}$$

where

A13
$$\kappa_{p}=\frac{Y_{\mathrm{tot}}K_{\mathrm{ads}}k_{\mathrm{rxn}}S_{\mathrm{tot}}}{1+K_{\mathrm{rxn}}Y_{\mathrm{tot}}}$$

The linearized expression for *P*(*t*) is only valid when *P*(*t*) ≤ *Y*_tot_. After that point, the amount of product cannot be assumed to be proportional to the gas-phase concentration. Thus, the maximum particle lifetime τ_max_ at which the *K*_sa,tot_ equilibrium model is applicable is

A14
$$\tau_{\max}=\frac{1+K_{\mathrm{rxn}}Y_{\mathrm{tot}}}{K_{\mathrm{ads}}k_{\mathrm{rxn}}S_{\mathrm{tot}}X_{\mathrm{gs}}}$$

### A2 Derivation of Parameters for Kinetic Aerosol Models

Used for kinetic modeling of heterogeneous uptake, the uptake coefficient γ is defined as the ratio of the net uptake flux of X by the surface to the total thermal collision flux of X with the surface:

A15
$$\gamma =\frac{k_{\mathrm{ads}}X_{\mathrm{gs}}\left(S_{\mathrm{tot}}-X_{s}\right)-k_{\mathrm{des}}X_{s}}{X_{\mathrm{gs}}\omega }$$

In a freshly emitted particle, uptake is fastest because none of the sorptive sites have been used yet, and this regime is characterized by initial uptake coefficient γ_0_, which can be derived by evaluating γ at *X*_s_ = 0:

A16
$$\gamma_{0}=\frac{k_{\mathrm{ads}}S_{\mathrm{tot}}}{\omega }$$

An expression for the uptake coefficient at quasi-steady-state γ_qss_ (after sorption has reached equilibrium, but while the reaction is still going) can be derived by evaluating γ at *X*_s,qss_ derived earlier:

A17
$$\gamma_{\mathrm{qss}}=\gamma_{0}\left[\frac{1+K_{\mathrm{ads}}X_{\mathrm{gs}}}{1+K_{\mathrm{ads}}X_{\mathrm{gs}}+K_{\mathrm{rxn}}\left(Y_{\mathrm{tot}}-P\right)}\right]$$

γ_qss_ depends on gas-phase concentration, but at atmospheric conditions, where concentrations are low, it can be approximated as a constant by performing a Taylor expansion around *X*_gs_ = 0 and keeping the zeroth-order term:

A18
$$\gamma_{\mathrm{qss}}\approx \gamma_{0}\left[\frac{K_{\mathrm{rxn}}\left(Y_{\mathrm{tot}}-P\right)}{1+K_{\mathrm{rxn}}\left(Y_{\mathrm{tot}}-P\right)}\right]$$

If we further make an assumption that the concentration of product *P* is close to zero, which is a valid assumption for aerosol particles over a large fraction of their lifetime, an unreacted-surface quasi-steady-state uptake coefficient γ_qss,ur_ can be derived:

A19
$$\gamma_{\mathrm{qss},\mathrm{ur}}=\gamma_{0}\frac{K_{\mathrm{rxn}}Y_{\mathrm{tot}}}{1+K_{\mathrm{rxn}}Y_{\mathrm{tot}}}$$

### A3 Time until Sorption Reaches Steady State

At low gas concentrations, sorption and reaction occur at different time scales and can be decoupled. To estimate the time when the transition between sorption-dominated and reaction-dominated uptake occurs, we write out and integrate the rate law for *X*_s_ with the assumptions that *X*_gs_ is constant and that the amount of product is constant and equal to zero (*P* = 0). The former assumption is valid at atmospheric conditions because the drop in gas concentration is very small, as seen in Figure 11a. The latter assumption is valid because the formation of the product is very slow at atmospheric conditions and the amount formed will be negligible at the time scale of sorption.

A20
$$\frac{dX_{s}}{dt}\approx k_{\mathrm{ads}}X_{\mathrm{gs}}\left(S_{\mathrm{tot}}-X_{s}\right)-k_{\mathrm{des}}X_{s}-k_{\mathrm{rxn}}X_{s}Y_{\mathrm{tot}}$$

Integration with the initial condition of zero yields an approximate expression for *X*_s_ as a function of time. Differentiation thereof produces a closed form algebraic expression for *dX*_s_/*dt*.

A21a
$$A:=k_{\mathrm{ads}}X_{\mathrm{gs}}S_{\mathrm{tot}}$$

A21b
$$B:=k_{\mathrm{ads}}X_{\mathrm{gs}}+k_{\mathrm{des}}+k_{\mathrm{rxn}}Y_{\mathrm{tot}}$$

A21c
$$X_{s}\approx \frac{A}{B}\left(1-e^{-Bt}\right)$$

A21d
$$\frac{dX_{s}}{dt}\approx Ae^{-Bt}$$

Sorption reaches steady state when *dX*_s_/*dt* ≈ 0. Since the derivative of *X*_s_ exhibits asymptotic behavior (eq 83), we will define the time when the system has reached steady state, *τ*_qss_, as the e-folding time of the derivative of *X*_s_. In other words, *τ*_qss_ is the time when *dX*_*s*_/*dt* has decreased by a factor of *e* from its original value, which according to eq 83 is equal to 1/*B*:

A22
$$\tau_{\mathrm{qss}}\approx \frac{1}{k_{\mathrm{ads}}X_{\mathrm{gs}}+k_{\mathrm{des}}+k_{\mathrm{rxn}}Y_{\mathrm{tot}}}$$

## Notes

Additional derivations, methods, and details needed for reproducibility (PDF)

## References

[1] Ravishankara A. R. (1997). Heterogeneous
and multiphase chemistry in the troposphere. Science.

[2] George I., Abbatt J. (2010). Heterogeneous oxidation of atmospheric aerosol particles
by gas-phase radicals. Nat. Chem..

[3] Cappa C. D., Che D. L., Kessler S. H., Kroll J. H., Wilson K. R. (2011). Variations
in organic aerosol optical and hygroscopic properties upon heterogeneous
OH oxidation. J. Geophys. Res.: Atmos..

[4] Jia X., Gu W., Peng C., Li R., Chen L., Wang H., Wang H., Wang X., Tang M. (2021). Heterogeneous reaction
of CaCO3 with NO2 at different relative humidities: Kinetics, mechanisms,
and impacts on aerosol hygroscopicity. J. Geophys.
Res.: Atmos..

[5] Zelenay V., Monge M. E., D’Anna B., George C., Styler S. A., Huthwelker T., Ammann M. (2011). Increased steady state uptake of
ozone on soot due to UV/Vis radiation. J. Geophys.
Res.: Atmos..

[6] Khalizov A. F., Cruz-Quinones M., Zhang R. (2010). Heterogeneous reaction of NO2 on
fresh and coated soot surfaces. J. Phys. Chem.
A.

[7] Winer A. M., Biermann H. W. (1994). Long pathlength
differential optical absorption spectroscopy
(DOAS) measurements of gaseous HONO, NO2 and HCNO in the California
South Coast Air Basin. Res. Chem. Intermed..

[8] Tham Y. J., Wang Z., Li Q., Wang W., Wang X., Lu K., Ma N., Yan C., Kecorius S., Wiedensohler A., Zhang Y., Wang T. (2018). Heterogeneous
N2O5 uptake coefficient
and production yield of ClNO2 in polluted northern China: roles of
aerosol water content and chemical composition. Atmos. Chem. Phys..

[9] Zhou W., Du H., Liu M., Xu W., Zhao J., Xie C., Wang Q., Zhang Z., Zhang Z., Li J. (2025). Role of N2O5 heterogeneous
hydrolysis in summer nitrate formation
in Beijing: insights from direct measurements and model simulations. Npj Clean Air.

[10] Thornton J. A., Kercher J. P., Riedel T. P., Wagner N. L., Cozic J., Holloway J. S., Dubé W. P., Wolfe G. M., Quinn P. K., Middlebrook A. M., Alexander B., Brown S. S. (2010). A large atomic chlorine
source inferred from mid-continental reactive nitrogen chemistry. Nature.

[11] Osthoff H. D., Roberts J. M., Ravishankara A. R., Williams E. J., Lerner B. M., Sommariva R., Bates T. S., Coffman D., Quinn P. K., Dibb J. E., Stark H., Burkholder J. B., Talukdar R. K., Meagher J., Fehsenfeld F. C., Brown S. S. (2008). High levels of nitryl chloride in the polluted subtropical
marine boundary layer. Nat. Geosci..

[12] Wang T., Tham Y. J., Xue L., Li Q., Zha Q., Wang Z., Poon S. C. N., Dubé W. P., Blake D. R., Louie P. K. K., Luk C. W. Y., Tsui W., Brown S. S. (2016). Observations of nitryl chloride and modeling its source
and effect on ozone in the planetary boundary layer of southern China. J. Geophys. Res.: Atmos..

[13] Solomon S., Garcia R. R., Rowland F. S., Wuebbles D. J. (1986). On the depletion
of Antarctic ozone. Nature.

[14] Webster C., May R., Toohey D., Avallone L., Anderson J., Newman P., Lait L., Schoeberl M., Elkins J., Chan K. (1993). Chlorine chemistry
on polar stratospheric cloud particles in the Arctic winter. Science.

[15] Tolbert M. A., Rossi M. J., Malhotra R., Golden D. M. (1987). Reaction
of chlorine
nitrate with hydrogen chloride and water at Antarctic stratospheric
temperatures. Science.

[16] Macintyre H., Evans M. (2010). Sensitivity of a global
model to the uptake of N2O5 by tropospheric
aerosol. Atmos. Chem. Phys..

[17] Evans M. J., Jacob D. J. (2005). Impact of new laboratory
studies of N2O5 hydrolysis
on global model budgets of tropospheric nitrogen oxides, ozone, and
OH. Geophys. Res. Lett..

[18] Riemer N., Vogel H., Vogel B., Schell B., Ackermann I., Kessler C., Hass H. (2003). Impact of the heterogeneous hydrolysis
of N2O5 on chemistry and nitrate aerosol formation in the lower troposphere
under photosmog conditions. J. Geophys. Res.:
Atmos..

[19] Khalizov A. F., Mao N. (2023). Heterogeneous reaction
of gaseous mercuric chloride with atmospherically
relevant organic films. ACS Earth Space Chem..

[20] Mao N., Antley J., Cooper M., Shah N., Kadam A., Khalizov A. (2021). Heterogeneous Chemistry
of Mercuric Chloride on Inorganic
Salt Surfaces. J. Phys. Chem. A.

[21] Lin C.-J., Pongprueksa P., Lindberg S. E., Pehkonen S. O., Byun D., Jang C. (2006). Scientific
uncertainties in atmospheric mercury models I: Model science
evaluation. Atmos. Environ..

[22] Malcolm E. G., Ford A. C., Redding T. A., Richardson M. C., Strain B. M., Tetzner S. W. (2009). Experimental investigation
of the
scavenging of gaseous mercury by sea salt aerosol. J. Atmos. Chem..

[23] Davidovits P., Kolb C. E., Williams L. R., Jayne J. T., Worsnop D. R. (2006). Mass accommodation
and chemical reactions at gas-liquid interfaces. Chem. Rev..

[24] Ammann M., Pöschl U., Rudich Y. (2003). Effects of reversible adsorption
and Langmuir-Hinshelwood surface reactions on gas uptake by atmospheric
particles. Phys. Chem. Chem. Phys..

[25] Shiraiwa M., Garland R. M., Pöschl U. (2009). Kinetic double-layer
model of aerosol
surface chemistry and gas-particle interactions (K2-SURF): Degradation
of polycyclic aromatic hydrocarbons exposed to O3, NO2, H2O, OH and
NO3. Atmos. Chem. Phys..

[26] Kroll J. H., Seinfeld J. H. (2008). Chemistry of secondary
organic aerosol: Formation and
evolution of low-volatility organics in the atmosphere. Atmos. Environ..

[27] Pankow J. F. (1987). Review
and comparative analysis of the theories on partitioning between the
gas and aerosol particulate phases in the atmosphere. Atmos. Environ..

[28] Rutter A. P., Schauer J. J. (2007). The effect of temperature on the
gas-particle partitioning
of reactive mercury in atmospheric aerosols. Atmos. Environ..

[29] Zhang D., Zhang R. (2005). Laboratory investigation
of heterogeneous interaction of sulfuric
acid with soot. Environ. Sci. Technol..

[30] Wang L., Lal V., Khalizov A. F., Zhang R. (2010). Heterogeneous chemistry of alkylamines
with sulfuric acid: Implications for atmospheric formation of alkylaminium
sulfates. Environ. Sci. Technol..

[31] Qiu C., Wang L., Lal V., Khalizov A. F., Zhang R. (2011). Heterogeneous
reactions of alkylamines with ammonium sulfate and ammonium bisulfate. Environ. Sci. Technol..

[32] Levitt N. P., Zhang R., Xue H., Chen J. (2007). Heterogeneous chemistry
of organic acids on soot surfaces. J. Phys.
Chem. A.

[33] Lal V., Khalizov A. F., Lin Y., Galvan M. D., Connell B. T., Zhang R. (2012). Heterogeneous reactions
of epoxides in acidic media. J. Phys. Chem.
A.

[34] Howard C.
J. (1979). Kinetic
measurements using flow tubes. J. Phys. Chem..

[35] Aubin D. G., Abbatt J. P. (2007). Interaction of NO2
with hydrocarbon soot: Focus on
HONO yield, surface modification, and mechanism. J. Phys. Chem. A.

[36] Pöschl U., Canagaratna M., Jayne J. T., Molina L. T., Worsnop D. R., Kolb C. E., Molina M. J. (1998). Mass accommodation coefficient of
H2SO4 vapor on aqueous sulfuric acid surfaces and gaseous diffusion
coefficient of H2SO4 in N2/H2O. J. Phys. Chem.
A.

[37] Gershenzon Y. M., Grigorieva V. M., Ivanov A. V., Remorov R. G. (1995). O3 and OH sensitivity
to heterogeneous sinks of HO x and CH3O2 on aerosol particles. Faraday Discuss..

[38] Knopf D. A., Pöschl U., Shiraiwa M. (2015). Radial diffusion and penetration
of gas molecules and aerosol particles through laminar flow reactors,
denuders, and sampling tubes. Anal. Chem..

[39] Brown R. (1978). Tubular flow
reactors with first-order kinetics. J. Res.
Natl. Bur. Stand.

[40] Cooney D. O., Kim S.-S., Davis E. J. (1974). Analyses of mass transfer in hemodialyzers
for laminar blood flow and homogeneous dialysate. Chem. Eng. Sci..

[41] Davis E. J. (2008). Interpretation
of uptake coefficient data obtained with flow tubes. J. Phys. Chem. A.

[42] Knopf D., Cosman L., Mousavi P., Mokamati S., Bertram A. (2007). A novel flow
reactor for studying reactions on liquid surfaces coated by organic
monolayers: Methods, validation, and initial results. J. Phys. Chem. A.

[43] Wilson K. R., Prophet A. M., Willis M. D. (2022). A kinetic model for predicting trace
gas uptake and reaction. J. Phys. Chem. A.

[44] Behr P., Terziyski A., Zellner R. (2004). Reversible gas adsorption in coated
wall flow tube reactors. Model simulations for Langmuir kinetics. Z. Phys. Chem..

[45] Cox R. A., Fernandez M. A., Symington A., Ullerstam M., Abbatt J. P. (2005). A kinetic model
for uptake of HNO3 and HCl on ice in
a coated wall flow system. Phys. Chem. Chem.
Phys..

[46] Bartels-Rausch T., Huthwelker T., Gäggeler H. W., Ammann M. (2005). Atmospheric pressure
coated-wall flow-tube study of acetone adsorption on ice. J. Phys. Chem. A.

[47] Hanson D., Ravishankara A. (1993). Uptake of hydrochloric acid and hypochlorous acid onto
sulfuric acid: Solubilities, diffusivities, and reaction. J. Phys. Chem..

[48] Pöschl U., Rudich Y., Ammann M. (2007). Kinetic model framework for aerosol
and cloud surface chemistry and gas-particle interactions - Part 1:
General equations, parameters, and terminology. Atmos. Chem. Phys..

[49] Ammann M., Pöschl U. (2007). Kinetic model framework for aerosol and cloud surface
chemistry and gas-particle interactions - Part 2: Exemplary practical
applications and numerical simulations. Atmos.
Chem. Phys..

[50] Sethuraman S., Westervelt D. M., Gong K., Grassian V. H., McNeill V. F. (2026). Modeling
and Parametrization of Size-Dependent Processes in Multiphase Aerosol
Chemistry. ACS ES&T Air.

[51] Willis M. D., Wilson K. R. (2022). Coupled interfacial
and bulk kinetics govern the timescales
of multiphase ozonolysis reactions. J. Phys.
Chem. A.

[52] Prophet A. M., Wilson K. R. (2025). A compartmentalized model of multiphase chemical kinetics. J. Chem. Phys..

[53] Yang D.-B., Chen X., Francisco J. S. (2026). Explicit
particle kinetics simulations
of reactive diffusion at air-water interfaces. J. Chem. Phys..

[54] Amos H. M., Jacob D. J., Holmes C. D., Fisher J. A., Wang Q., Yantosca R. M., Corbitt E. S., Galarneau E., Rutter A. P., Gustin M. S., Steffen A., Schauer J. J., Graydon J. A., St. Louis V. L., Talbot R. W., Edgerton E. S., Zhang Y., Sunderland E. M. (2012). Gas-particle
partitioning of atmospheric
Hg­(II) and its effect on global mercury deposition. Atmos. Chem. Phys..

[55] Wu L., Mao H., Ye Z., Dibble T., Saiz-Lopez A., Zhang Y. (2024). Improving simulation of gas-particle partitioning of atmospheric
mercury using CMAQ-newHg-Br v2. J. Adv. Model.
Earth Syst..

[56] Murphy D. M., Fahey D. W. (1987). Mathematical treatment of the wall loss of a trace
species in denuder and catalytic converter tubes. Anal. Chem..

[57] COMSOL AB. COMSOL Multiphysics, Version 6.2 https://www.comsol.com. accessed 21 July 2026.

[58] Cohen S. D., Hindmarsh A. C., Dubois P. F. (1996). CVODE, a stiff/nonstiff ODE solver
in C. Comput. Phys..

[59] Hindmarsh A. C., Brown P. N., Grant K. E., Lee S. L., Serban R., Shumaker D. E., Woodward C. S. (2005). SUNDIALS:
Suite of nonlinear and
differential/algebraic equation solvers. ACM
Trans. Math. Software.

[60] Agarwal, S. ; Mierle, K. Ceres Solver version 2.2; http://ceres-solver.org. accessed 21 July 2026.

[61] Demidov, E. CSTR Model Standalone Implementation; https://github.com/egor-demidov/0d_adsorption. accessed 21 July 2026.

[62] Demidov, E. Heterogeneous Uptake Model Web Application; https://www.edemidov.com/heterogeneous-uptake. accessed 21 July 2026.

[63] Rutter A.
P., Schauer J. J. (2007). The impact
of aerosol composition on the particle to
gas partitioning of reactive mercury. Environ.
Sci. Technol..

[64] Langmuir I. (1918). The evaporation
of small spheres. Phys. Rev..

[65] Seinfeld, J. H. ; Pandis, S. N. Atmospheric chemistry and physics: from air pollution to climate change, 3rd ed.; John Wiley & Sons, 2016.

[100] Shah V., Jacob D. J., Thackray C. P., Wang X., Sunderland E. M., Dibble T. S., Saiz-Lopez A., Černušák I., Kellö V., Castro P. J., Wu R., Wang C. (2021). Improved mechanistic
model of the atmospheric redox chemistry of mercury. Environ. Sci. Technol..

[66] Luippold A., Gustin M. S., Dunham-Cheatham S. M., Zhang L. (2020). Improvement of quantification
and identification of atmospheric reactive mercury. Atmos. Environ..

[67] Miller M. B., Howard D. A., Pierce A. M., Cook K. R., Keywood M., Powell J., Gustin M. S., Edwards G. C. (2021). Atmospheric reactive
mercury concentrations in coastal Australia and the Southern Ocean. Sci. Total Environ..

[68] Gustin M. S., Dunham-Cheatham S. M., Allen N., Choma N., Johnson W., Lopez S., Russell A., Mei E., Magand O., Dommergue A., Elgiar T. (2023). Observations of the chemistry and
concentrations of reactive Hg at locations with different ambient
air chemistry. Sci. Total Environ..

[69] Holmes C. D., Jacob D. J., Corbitt E. S., Mao J., Yang X., Talbot R., Slemr F. (2010). Global atmospheric
model for mercury
including oxidation by bromine atoms. Atmos.
Chem. Phys..

