# Supplementary material for: A Model to Derive Langmuir–Hinshelwood Parameters from Heterogeneous Uptake Experiments in a Fast Flow Reactor
Source: J Phys Chem A. 2026 Aug 27;130(36):7232–52. doi: 10.1021/acs.jpca.6c03212 (PMC13573623; doi:10.1021/acs.jpca.6c03212)
Supplement: Supplementary file 1 [file jp6c03212_si_001.pdf]

A Model to Derive Langmuir-Hinshelwood Parameters from Heterogeneous Uptake Experiments in a Fast Flow Reactor

Egor Demidov[1,‡] and Alexei Khalizov[1,§]

[1]Department of Chemistry and Environmental Science,
New Jersey Institute of Technology, Newark, NJ 07102
[‡]Corresponding author: ed242@njit.edu
[§]Corresponding author: khalizov@njit.edu

S1 Sensitivity ODEs

In this section, we derive the sensitivity ODEs for the augmented system of equations for the chained CSTR model. First, let us remember the system of ODEs that defines the chained CSTR model:

$$\frac{dX_i}{dt} = \frac{FN_{\text{react}}}{\pi R^2 L}\left(X_{i-1} - X_i\right) - k_{\text{diff}}\left(X_i - X_{\text{gs},i}\right) \tag{S1a}$$

$$\frac{dX_{\text{gs},i}}{dt} = k_{\text{diff}}\left(X_i - X_{\text{gs},i}\right) - \frac{2}{R}k_{\text{ads}}f_{\text{sw}}(t)X_{\text{gs},i}(S_{\text{tot}} - X_{\text{s},i}) + \frac{2}{R}k_{\text{des}}X_{\text{s},i} \tag{S1b}$$

$$\frac{dX_{\text{s},i}}{dt} = k_{\text{ads}}f_{\text{sw}}(t)X_{\text{gs},i}(S_{\text{tot}} - X_{\text{s},i}) - k_{\text{des}}X_{\text{s},i} - k_{\text{rxn}}X_{\text{s},i}(Y_{\text{tot}} - P_i) \tag{S1c}$$

$$\frac{dP_i}{dt} = k_{\text{rxn}}X_{\text{s},i}(Y_{\text{tot}} - P_i) \tag{S1d}$$

Let the physical states in reactor i be represented by a state vector $\mathbf{x}_i$ and let the parameters of the Langmuir-Hinshelwood mechanism be represented by a parameter vector $\boldsymbol{\theta}$:

$$\mathbf{x}_i = \begin{bmatrix} X_i \\ X_{\text{gs},i} \\ X_{\text{s},i} \\ P_i \end{bmatrix} \quad \boldsymbol{\theta} = \begin{bmatrix} k_{\text{ads}} \\ k_{\text{des}} \\ k_{\text{rxn}} \\ S_{\text{tot}} \\ Y_{\text{tot}} \end{bmatrix} \tag{S2}$$

The derivative of state vector $\mathbf{x}_i$ with respect to time, $\dot{\mathbf{x}}_i$, is the system of ODEs given in Equation S1. Let a shorthand for the system be a vector-valued function $\mathbf{f}_i$, which depends on the state vector in the current reactor, the state vector in the previous reactor (because of the effluent stream coupling in Equation S1a), the parameter vector, and time:

$$\dot{\mathbf{x}}_i = \mathbf{f}_i(\mathbf{x}_i, \mathbf{x}_{i-1}, \boldsymbol{\theta}, t) \tag{S3}$$

Let us define the sensitivity matrix for reactor i as:

$$\mathbf{S}_i(t) := \frac{\partial \mathbf{x}_i}{\partial \boldsymbol{\theta}} \tag{S4}$$

Then the derivative of $\mathbf{S}_i$ with respect to time can be evaluated by differentiating $\mathbf{f}_i$ with respect to $\boldsymbol{\theta}$, since the order of differentiation of continuous second-order partial derivatives does not matter (Clairaut's Theorem):

$$\frac{\partial}{\partial t}\left(\frac{\partial \mathbf{x}_i}{\partial \boldsymbol{\theta}}\right) = \frac{\partial}{\partial \boldsymbol{\theta}}\mathbf{f}_i(\mathbf{x}_i, \mathbf{x}_{i-1}, \boldsymbol{\theta}, t) \tag{S5}$$

Since $\mathbf{x}_i$ and $\mathbf{x}_{i-1}$ both depend on θ, chain rule needs to be applied:

$$\frac{\partial}{\partial \boldsymbol{\theta}}\mathbf{f}_i(\mathbf{x}_i, \mathbf{x}_{i-1}, \boldsymbol{\theta}, t) = \frac{\partial \mathbf{f}_i}{\partial \mathbf{x}_i}\frac{\partial \mathbf{x}_i}{\partial \boldsymbol{\theta}} + \frac{\partial \mathbf{f}_i}{\partial \mathbf{x}_{i-1}}\frac{\partial \mathbf{x}_{i-1}}{\partial \boldsymbol{\theta}} + \frac{\partial \mathbf{f}_i}{\partial \boldsymbol{\theta}} \tag{S6}$$

Thus, the sensitivity ODEs have been derived:

$$\dot{\mathbf{S}}_i = \frac{\partial \mathbf{f}_i}{\partial \mathbf{x}_i}\mathbf{S}_i + \frac{\partial \mathbf{f}_i}{\partial \mathbf{x}_{i-1}}\mathbf{S}_{i-1} + \frac{\partial \mathbf{f}_i}{\partial \boldsymbol{\theta}} \tag{S7}$$

With 4 states and 5 parameters, $\mathbf{S}_i$ is a 4×5 matrix with element $(S_i)_{mn}$ corresponding to the sensitivity of state $(x_i)_m$ with respect to parameter θ_n.

The effect of varying each fitted parameter on simulated uptake curves is illustrated in Figure S1.

S2 Estimation of standard errors

Standard errors of fitted parameters were estimated from variance of the data and the Jacobian matrix:

$$\sigma^2 = \frac{S_{\text{sr}}(\boldsymbol{\theta})}{N-5} \tag{S8}$$

where σ^2 is the noise variance, N is the number of points, and $S_{\text{sr}}(\boldsymbol{\theta})$ is the sum of squared residuals at the parameter vector $\boldsymbol{\theta}$. The parameter variance matrix $\mathbf{C}$ was then computed from the Jacobian $\mathbf{J}$ of X with respect to parameters $\boldsymbol{\theta}$:

$$\mathbf{C} = \sigma^2\left(\mathbf{J}^T\mathbf{J}\right)^{-1} \tag{S9}$$

For N time points and 5 parameters, $\mathbf{J}$ is an $N \times 5$ matrix with elements given by:

$$J_{ji} = \frac{\partial X(t_j)}{\partial \theta_i} \tag{S10}$$

where $X(t_j)$ is outlet concentration evaluated a time t_j out of N time points. The standard error $E_{\text{std},i}$ of parameter θ_i was then computed from $\mathbf{C}$:

$$E_{\text{std},i} = \sqrt{C_{ii}} \tag{S11}$$

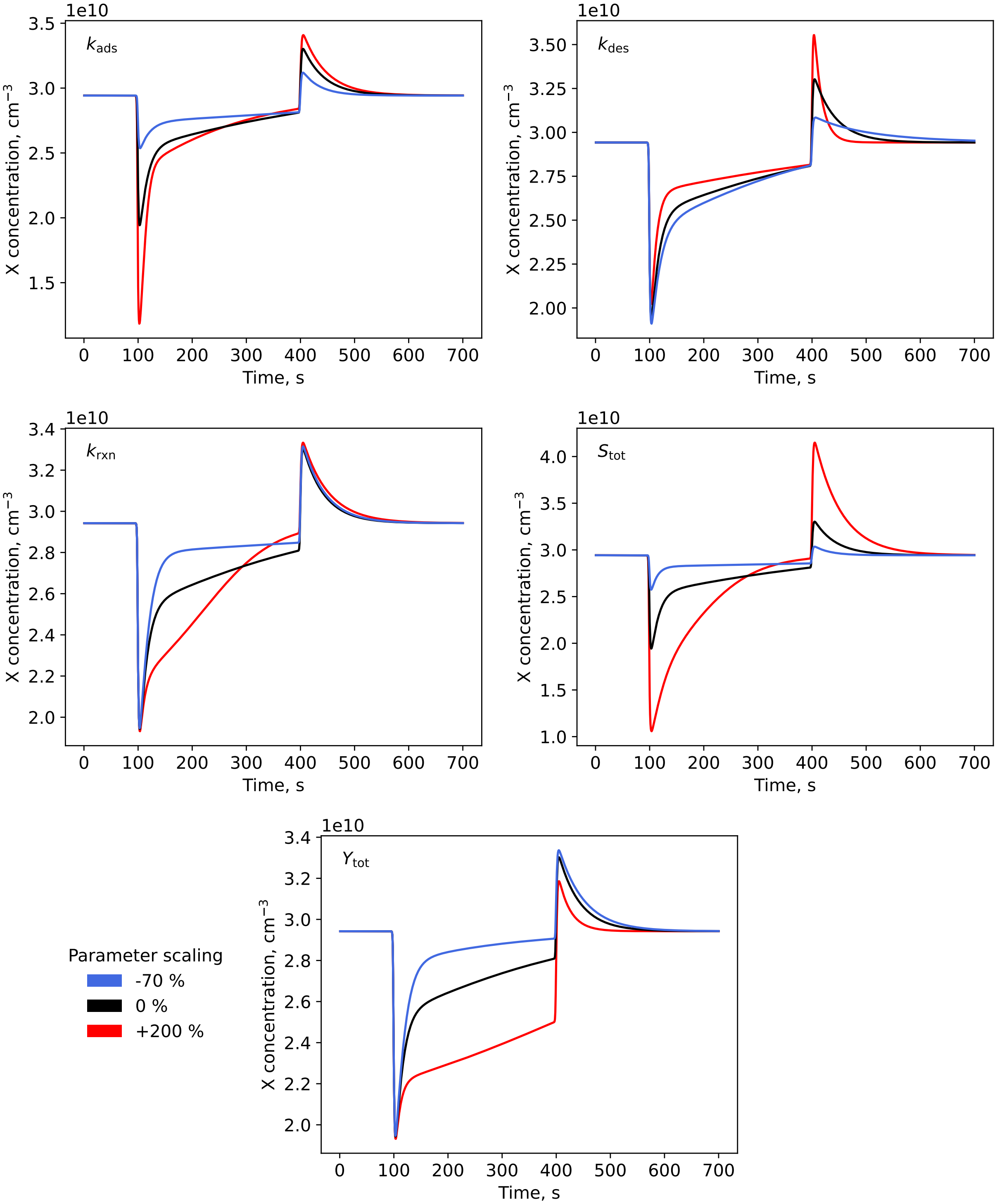

Figure S1: The effect of scaling each fitted parameter up or down on the overall shape of simulated uptake curves

S3 Artificial noise generation

To generate simulated experimental data for the purpose of analysis of optimal conditions for retrieval of k_{rxn} and Y_{tot}, three types of noise were added to each simulated curve: cumulative noise, low-frequency random noise, and high-frequency random noise. The numerical procedure used to generate noise and parameters that characterize the noise are provided in this section.

Let the concentration time-series generated with a model run be stored in a vector $\mathbf{X}$ of size N and let generated noise be stored in vector $\boldsymbol{\sigma}$ of size M where $M \leq N$. Let us define noise frequency ν, which has units of number of noise points per concentration point:

$$\nu := \frac{M}{N} \tag{S12}$$

Depending on the type of noise, $\boldsymbol{\sigma}$ is generated differently. For random noise, element j of $\boldsymbol{\sigma}_{\mathrm{rand}}$ is given by:

$$\sigma_{\mathrm{rand},j} = \alpha\epsilon_j, \qquad \epsilon_j \sim \mathcal{N}(0,1) \tag{S13}$$

where α is the noise magnitude parameter and ϵ_j is a random number sampled from a standard normal distribution. For cumulative noise, element j of $\boldsymbol{\sigma}_{\mathrm{cum}}$ is given by:

$$\sigma_{\mathrm{cum},j} = \sigma_{\mathrm{cum},j-1} + \alpha\epsilon_j, \qquad \epsilon_j \sim \mathcal{U}(-1,1) \tag{S14}$$

where α is the noise magnitude parameter and ϵ_i is a random number sampled from a uniform distribution ranging from -1 to 1.

Table S1 provides the parameters used for low-frequency random noise $\boldsymbol{\sigma}_{\mathrm{rand,lf}}$, high-frequency random noise $\boldsymbol{\sigma}_{\mathrm{rand,hf}}$, and cumulative noise $\boldsymbol{\sigma}_{\mathrm{cum}}$:

Table S1: Parameters used to generate the three noise arrays

Noise	α	ν
$\boldsymbol{\sigma}_{\mathrm{rand,lf}}$	2×10^8	0.33
$\boldsymbol{\sigma}_{\mathrm{rand,hf}}$	1×10^8	1.0
$\boldsymbol{\sigma}_{\mathrm{cum}}$	1×10^8	0.33

Before addition of noise arrays to the concentration array, noise arrays were interpolated to match the length of the concentration array N. Interpolation was performed such that the first and last points of a noise array were aligned with the first and last points of the concentration array.

Finally, the simulated experimental concentration array $\tilde{\mathbf{X}}$ was computed by adding the three noise arrays to the simulated uptake curve $\mathbf{X}$:

$$\tilde{\mathbf{X}} = \mathbf{X} + \boldsymbol{\sigma}_{\mathrm{rand,lf}} + \boldsymbol{\sigma}_{\mathrm{rand,hf}} + \boldsymbol{\sigma}_{\mathrm{cum}} \tag{S15}$$

S4 Experimental conditions for analyzed uptake curves

Experimental conditions and miscellaneous non-fitted parameters that were used in model runs to obtain fits discussed in the main text are shown in Table S2.

Table S2: Experimental conditions and other model inputs used to obtain fits discussed in main text

Parameter	NaCl	levoglucosan	Unit
F_{st}	2.493	2.368	$\mathrm{cm}^3\ \mathrm{s}^{-1}$
R	0.78	0.78	cm
L_{st}	2	5	cm
$D_{\mathrm{g,st}}$	0.431	0.431	$\mathrm{cm}^2\ \mathrm{s}^{-1}$
N_{react}	20	20	—
P	1.965	1.924	Torr
t_{start}	267.24	157.88	s
t_{end}	543.78	293.28	s
$\tau_{\mathrm{sw,1}}$	1	1	s
$\tau_{\mathrm{sw,2}}$	2.5	8.5	s

S5 Preprocessing of experimental data

Experimental data was preprocessed before fitting to correct for drift in baseline signal. An illustration of the drift correction procedure is shown in Figure S2. Drift correction was performed by traversing an uptake curve from left to right and adding points to the curve at times when the curve should have been at the baseline. At the time a point was added, a correction factor equal to the shift needed to be applied to the curve to bring it to the baseline concentration was computed at the added point. The uptake curve between the point that was just added and the previous point was then shifted by a linear function, which was equal zero at the previous point and to the correction factor at the current point. The uptake curve to the right of the current point was shifted by the correction factor.

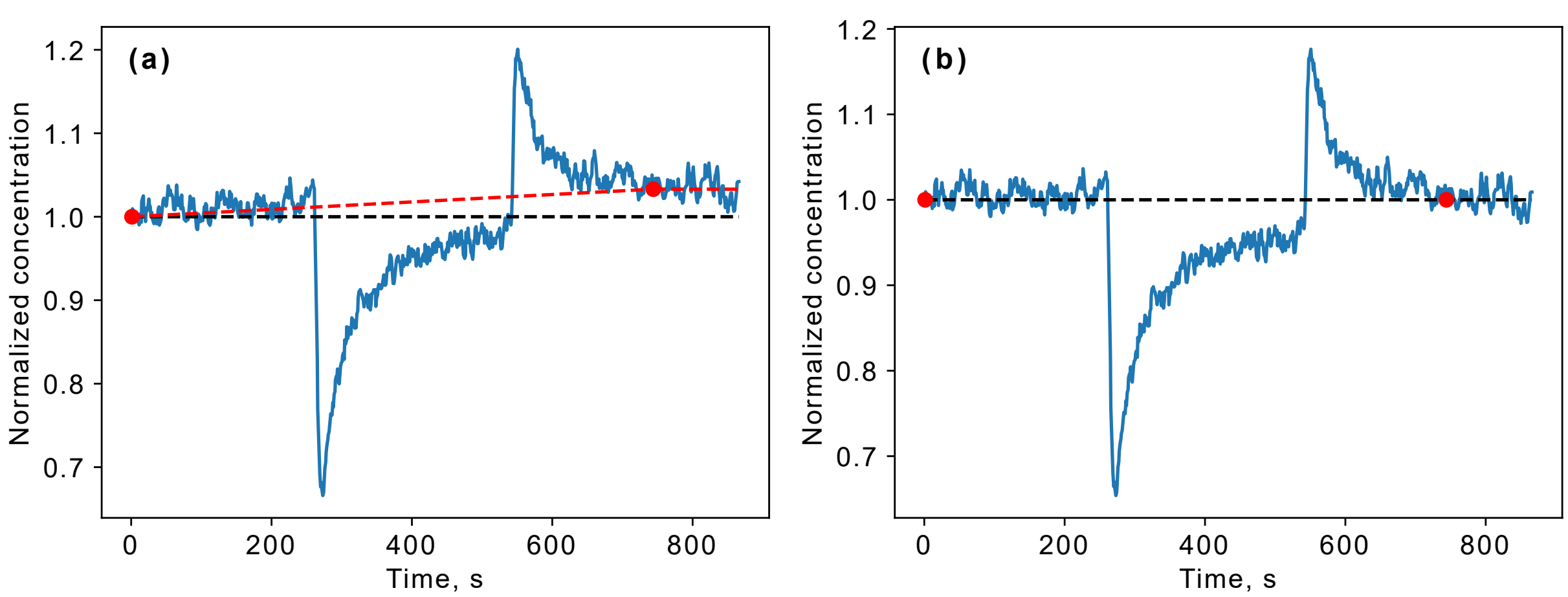

Figure S2: Illustration of preprocessing steps applied to experimental uptake curves. (a) Data before application of drift correction (red dashed curve indicates the correction function, black dashed curve indicates the baseline) and (b) data after application of drift correction (red dashed curve was subtracted from the blue curve)
